# Investigation of the Cross-talk Mechanism in Caco-2 Cells during *Clostridium difficile* Infection through Genetic-and-Epigenetic Interspecies Networks: Big Data Mining and Genome-Wide Identification

**DOI:** 10.3389/fimmu.2017.00901

**Published:** 2017-08-02

**Authors:** Cheng-Wei Li, Ming-He Su, Bor-Sen Chen

**Affiliations:** ^1^Laboratory of Control and Systems Biology, National Tsing Hua University, Hsinchu, Taiwan

**Keywords:** *Clostridium difficile*, Caco-2 cells, *Clostridium difficile* infection, genetic-and-epigenetic interspecies network, cross-talk molecular mechanism, reactive oxygen species, endoplasmic reticulum stress

## Abstract

*Clostridium difficile* is the leading cause of nosocomial antibiotic-associated diarrhea and the major etiologic agent of pseudomembranous colitis. In severe cases, *C. difficile* infection (CDI) can cause toxic megacolon, intestinal perforation, and death. The intestinal epithelium is the first tissue encountered in the adhesion and colonization of *C. difficile*, and serves as a physical defense barrier against infection. Despite the well-characterized cytotoxicity, few studies have investigated the genome-wide interplay between host cells and *C. difficile*. The aim of this study is to investigate the genetic-and-epigenetic molecular mechanisms between human colorectal epithelial Caco-2 cells and *C. difficile* during the early (0–60 min) and late stages (30–120 min) of infection. To investigate the cross-talk mechanisms during the progression of infection, we introduced a systems biology approach using big data mining, dynamic network modeling, a genome-wide data identification method, system order detection scheme, and principal network projection method (PNP). We focused on the construction of genome-wide genetic-and-epigenetic interspecies networks (GEINs) and subsequent extraction of host–pathogen core networks (HPNs) to investigate the progression of underlying host/pathogen genetic-and-epigenetic mechanisms from the early to late stages of CDI. Based on our results, we suggest that the cell-wall proteins CD2787 and CD0237, which both play an important role in cell adhesion and pathogen defense mechanisms, can be considered as potential drug targets. In addition, the crucial proteins employed by *C. difficile* for sporulation, including CD1214, CD2629, and CD2643, can also be considered as potential drug targets since spore-mediated re-infection is a critical issue.

## Introduction

*Clostridium difficile* (*C. difficile*) is characterized as the major infectious cause of antibiotic-associated diarrhea and is the etiologic agent of pseudomembranous colitis. It was first identified by Hall and O’Toole in 1935, but no further studies linked the bacterium to human disease until 1978 (1, 2). *C. difficile* infection (CDI) usually occurs after a disturbance of the normal gut microbiome following antibiotic treatment. After the disruption of the microbiota, *C. difficile* can colonize to intestinal epithelial cells and produce pathogenic factors to breach the barrier. The major toxins of *C. difficile* are enterotoxin CD0663 (TcdA) and cytotoxin CD0660 (TcdB). Both toxins enter host cells *via* receptor-mediated endocytosis and are cytotoxic to host tissue by inactivating small Rho GTPases (RAC1, RHOA, and CDC42) (3). The glucosylation-dependent inactivation of Rho GTPases results in actin cytoskeleton depolymerization and tight junction breakdown. However, *C. difficile* has not been fully investigated due to difficulties in its genetic manipulation, which makes it hard to generate isogenic strains for further study. In a previous study, the glucosylation of RHOA was shown to achieve saturation at 60 min postinfection, and all GTPases (CDC42, RAC1, and RHOA) also lose enzyme activity at this time point (4, 5). In addition, the MTT-dependent cell viability assays presented in our data source study (6) reveal that significant cell death also occurs at 60 min postinfection. Based on these observations, we define the early (0–60 min) and later stages (30–120 min) of host cells in CDI to investigate the progression of molecular mechanisms between two species. The 30-min overlap allows us to observe the causality and coherence of cross-talk molecular mechanisms.

Another mechanism regulating colonic gene expression is the microRNA (miRNA) system. miRNAs are small non-coding RNA molecules (~22 nucleotides) that bind to mRNA *via* complementary base pairing, resulting in mRNA silencing in human cells. Interestingly, a recent study indicates that the host utilizes miRNA silencing to shape the gut microbiota, including the Clostridium genus (7), suggesting that miRNAs play a crucial role in not only host gene repression but also microbiota shaping.

Unlike miRNAs, long non-coding RNAs (lncRNAs) are too large (~200–1,000 nucleotides) to pass through the bacterium cell wall and cell membrane for pathogen-gene regulation. In human cells, lncRNAs participate in gene regulation in a similar but more complex manner than miRNA (8), controlling various cellular responses. Furthermore, other epigenetic regulations, such as DNA methylation and histone modification, confer rapid and strong cellular responses to bacterial invasion. In order to investigate the progression of host–pathogen cross-talk mechanisms, as well as how these epigenetic activities contribute to progression during CDI, we identified the genome-wide genetic-and-epigenetic interspecies networks (GEINs) in both the host and pathogen during the early and late stages of CDI. We then extracted the host–pathogen core networks (HPNs) from the GEINs to investigate the core pathways involved in the cellular responses of the host and pathogen during the early and late stages of CDI. In addition, we also discussed the offensive and defense mechanisms employed by the host and pathogen, respectively.

## Results

### The Identified GEINs at the Early and Late Stage of CDI

**Figure 1:**
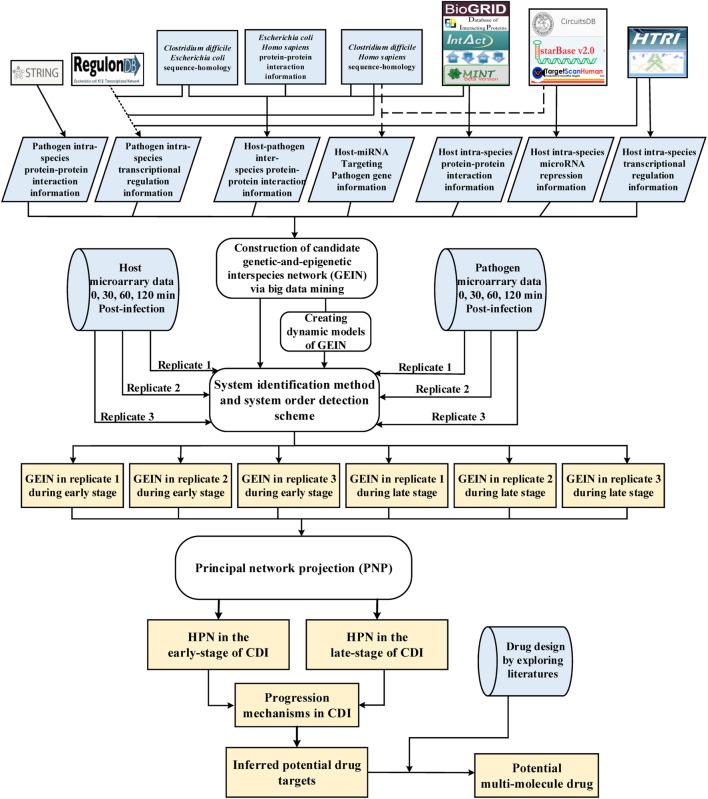
Flowchart of the systems biology approach used to construct genetic-and-epigenetic interspecies networks (GEINs) for host–pathogen core networks (HPNs) and to investigate the cross-talk mechanisms during *C. difficile* infection (CDI) for drug targets. The blue gray blocks represent the external information utilized in this study, including the big data mining for constructing candidate genetic-and-epigenetic interspecies network (GEIN), microarray data identification, and the surveyed literature for drug design; the rounded rectangular blocks denote the schemes and methods utilized to construct the candidate GEIN and real cross-talk GEINs at the early and late stages of CDI, and then extract the HPNs of each stage; and the light yellow blocks are the identified real GEINs and the cross-talk HPNs at the early and late stages of infection, as well as the inferred drug targets.

**Figure 2:**
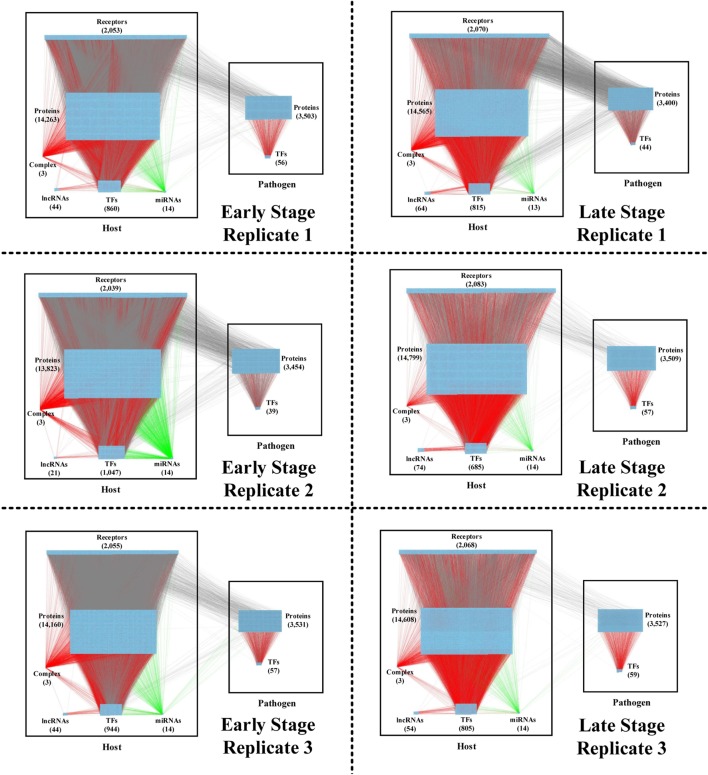
The real genetic-and-epigenetic interspecies networks (GEINs) of three replicates during the early and late stages of *C. difficile* infection (CDI). These figures show that the identified real genome-wide GEINs of each replicate during the early and late stages of infection. The gray lines represent protein–protein interaction; the red lines denote the transcriptional regulation; and the green lines signify the microRNA (miRNA) repression.

By applying a system identification method and system order detection scheme to two-sided microarray data (see materials and methods, supplementary methods and the flowchart in Figure 1), we identified the early-stage and late-stage GEINs of three biological replicates using the network visualizing software Cytoscape (9) (Figure 2). The numbers of identified nodes and edges are also shown in Tables 1 and 2, respectively. Among all three replicates, the node number of host transcription factors (TFs) at the early stage is higher than that at the late stage. In addition, the identified edges in Table 2 show significant differences in host-TFs to host-genes regulation between the two stages. These results reveal that the activities of host TFs are more abundant during the early stage.

**Table 1:** The number of identified nodes in the early stage and late stage of each replicate.

Nodes	Candidates	E_R1	L_R1	E_R2	L_R2	E_R3	L_R3
HP	14,686	14,263	14,565	13,823	14,799	14,160	14,608
HR	2,137	2,053	2,070	2,039	2,083	2,055	2,068
HT	1,780	860	815	1,047	685	944	805
HM	14	14	13	14	14	14	14
HL	223	44	64	21	74	44	54
HC	3	3	3	3	3	3	3
PP	3,585	3,503	3,400	3,454	3,509	3,531	3,527
PT	71	56	44	39	57	57	59
Total nodes	22,499	20,796	20,974	20,440	21,224	20,808	21,138

*E_R1 and L_R1 represent the early stage and late stage of replicate 1, respectively. Similarly, E_R2 and L_R2 denote the early stage and late stage of replicate 2, and E_R3 and L_R3 are the early stage and late stage of replicate 3. HP denotes host cytosolic protein [excluding host receptor and host transcription factor (TF)]; HR, HT, HM, HL, and HC represent host receptor, host TF, host microRNA, host long non-coding RNA and host complex, respectively; PP means pathogen protein excluding pathogen TF; and PT denotes pathogen TF*.

**Table 2:** The number of identified edges in the early stage and late stage of each replicate.

Edges	Candidates	E_R1	L_R1	E_R2	L_R2	E_R3	L_R3
HT →HG	138,486	20,800	18,594	26,695	16,483	21,770	18,827
HT →HM	31	17	6	4	6	7	8
HT →HL	218	47	76	34	90	55	64
HL →HG	184	12	3	27	8	7	15
HM →HG	5,961	357	165	1,163	176	379	198
HC →HG	5,442	1,171	1,304	1,826	347	1,175	738
HM →PP	96	4	5	0	2	19	7
PT →PG	1,265	543	449	262	526	671	619
HG—HG	3,425,976	109,102	41,123	183,116	29,978	104,647	34,759
HG—PG	17,068	610	2,264	1,333	226	544	145
PG—PG	290,018	9,826	36,279	41,960	7,543	14,202	3,989
Total edges	3,884,745	142,489	100,268	256,420	55,385	143,476	59,369

*The arrow lines in first column represent transcriptional/post-transcriptional regulations; the solid lines in the first column denote protein–protein interactions; HG signify host genes; and PG are pathogen genes*.

To further characterize genes in Caco-2 cells according to their functional groups, we performed an enrichment analysis *via* the function annotation tool DAVID (10) on the conserved target genes among all three replicates based on the biological process categories of GO database and the protein information resources of the Swiss-Prot database (Table 3). The early stage of CDI was characterized by the disturbance of cell shape and epithelial cell barrier, as well as immune activation and metal binding, which plays an important role in the scramble for metallic nutrients between the host and pathogen. At the late stage, the results including inflammatory-related functions and molecule secretion/transport suggests that a strong inflammatory response is triggered to eliminate the pathogen.

**Table 3:** The functional enrichment analysis of the conserved target genes among three replicates based on GO terms and protein information resources of Swiss-Prot.

Category	Term	*p-Value*
**Early stage**
SP_PIR_KEYWORDS	Metal binding	5.73E−05
SP_PIR_KEYWORD	Disease mutation	0.002178622
GOTERM_BP_FAT	GO:0048858~cell projection morphogenesis	0.004720934
GOTERM_BP_FAT	GO:0002252~immune effector process	0.021024346
SP_PIR_KEYWORDS	Tight junction	0.04930793
**Late stage**
SP_PIR_KEYWORDS	Secreted	8.84E−04
GOTERM_BP_FAT	GO:0042116~macrophage activation	0.002396512
GOTERM_BP_FAT	GO:0006954~inflammatory response	0.007684734
GOTERM_BP_FAT	GO:0051181~cofactor transport	0.009323758
GOTERM_BP_FAT	GO:0006910~phagocytosis, recognition	0.040000612

*The early stage of *C. difficile* infection is characterized by the change of cell shape and tight junction and this could result from the activities of GTPases and pathogen toxins. The metal-binding ability is crucial for both host and pathogen cells due to its important role in the scramble of metallic nutrients and the transport of toxic molecule, including ROS. Signaling in the case of the early stage of infection was increased for immune response, while signaling in the case of the late stage of infection was increased for the abundant cellular processes, including macrophage activation, phagocytosis, and inflammatory response. The cofactor transport and secretion-related process also contribute to the cytokine production and secretion*.

Since GEINs are very complex, it is difficult to investigate the precise host–pathogen interaction process from these networks directly. We, therefore, performed the principal network projection method (PNP) method to extract the core nodes with high projection values, which compose the corresponding HPNs from early-stage and late-stage GEINs of Caco-2 cells during CDI.

### The HPNs during the Infection of *C. difficile*

#### Construction of HPNs to Investigate the Epigenetic Activities in Host Core Networks of CDI

Applying the PNP method to GEINs in Figure 2, host/pathogen proteins with top 2,000 projection values based on intraspecies ranking in all three replicates and their connected genes/miRNAs/lncRNAs/complex were selected as core nodes of GEINs of each stage. Since the identified GEINs in Figure 2 belong to three biological replicates from the same cell line, the identified differential interactions and regulations can be viewed as the adaptability of cells while facing stress and stimulus at different replicates. For more complete information, the combinations of these interactions/regulations in three replicates are considered real GEINs in the early and late stages as shown in Figures S1 and S2 in Supplementary Material, respectively. Next, we extracted core nodes from the real GEINs in Figures S1 and S2 in Supplementary Material using the PNP method to consist HPNs as shown in Figures S3 and S4 in Supplementary Material at the early and the late stages, respectively.

In order to adapt to CDI, some post-translation epigenetic modifications in host cells can also be found in HPNs. These epigenetic modifications can be detected by the basal level $\kappa_{i}^{H}$ in the host protein expression dynamic Eq. 1 in the Section “MATERIALS AND METHODS.” During the early stage of CDI (Figure S3 in Supplementary Material), host MAPK pathway members (UBA52 and HSPA5) can be regulated by the deacetylase protein (HDAC11) and the ubiquitin protein (UBE2D3). In addition, the host proteins (DUSP6 and EGFR) involved in the interleukin-3, -5 signaling pathway, can also be regulated by the methyltransferase protein (PRDM14), the deubiquitinase protein (OTUB1), and the ubiquitin protein (UCHL5). These activities of the MAPK pathway and interleukin-related pathway participate in the immune response of host cells in response to the invasive bacterium. This finding can be supported by the functional enrichment analysis of early stage GEIN in Table 3. Furthermore, in Figure S3 in Supplementary Material, the small GTPase (CDC42) and downstream effectors (GRB2 and KBTBD7) that participate in the GTPase signaling pathway could be regulated by the deacetylase protein (HDAC4) and ubiquitin proteins (UBA52, USP43). In addition to post-translation modification, our results show that host heat shock protein (HSP) HSPA5 is DNA methylated in the HPN at the early stage of CDI. Considering that host–pathogen interspecies interactions play a central role in bacterial invasion, we then aim to investigate the cross talk between *C. difficile* proteins and host plasma membrane proteins.

#### Cross-talk Networks among Host–Pathogen Interactions and Their Validations

**Figure 3:**
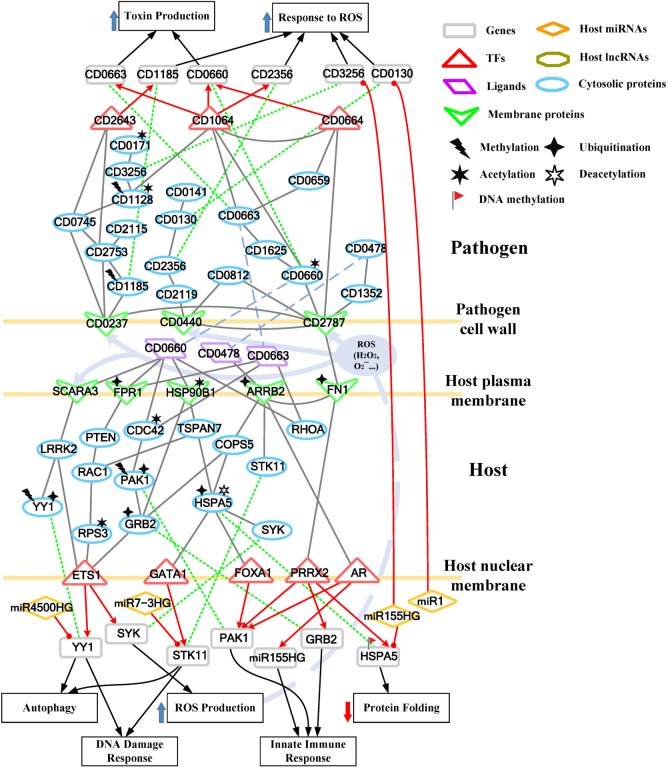
Core pathways rearranged from the host–pathogen core network in Figure S3 in Supplementary Material at the early stage of *C. difficile* infection (CDI). The upper clump represents the pathogen core pathways and the lower clump signifies the host core pathways at the early stage of CDI. The gray solid lines denote the protein–protein interaction; the red arrow lines are transcriptional regulation; the green dot lines signify protein translation; the gray blue dash lines represent protein secretion; and the purple clump with arrow and dash lines indicate the activity of reactive oxygen species (ROS). The pathogenic factors (CD0660, CD0663, and CD0478) of *C. difficile* trigger ROS production and dysfunction of protein folding of Caco-2 cells. Therefore, host cells employ autophagy and DNA damage response to remove induced cellular injuries and activate the immune response to eliminate pathogen. In response, *C. difficile* generate various antioxidative proteins to counteract the host-produced ROS.

**Figure 4:**
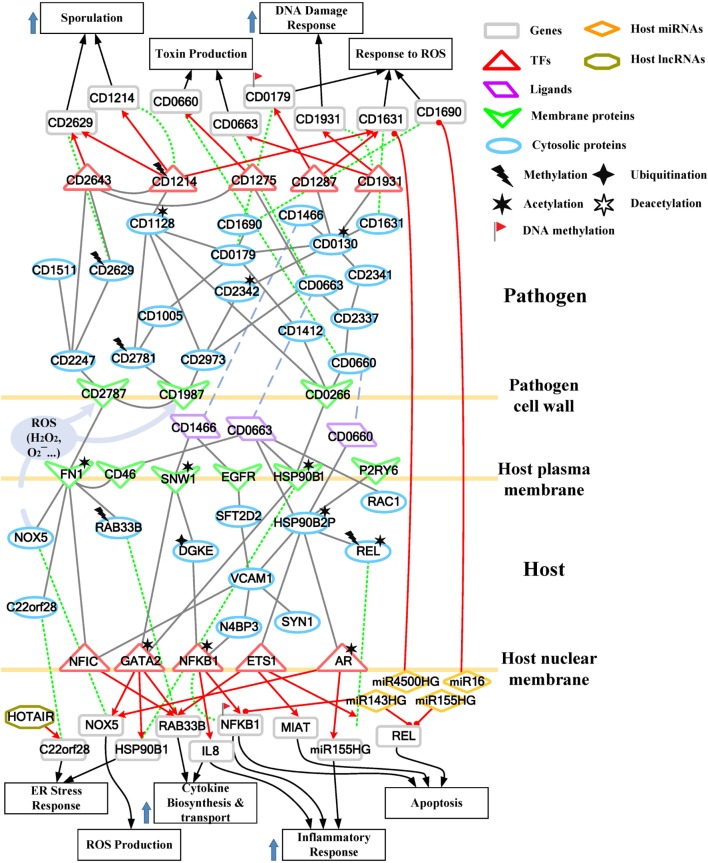
Core pathways rearranged from the host–pathogen core network in Figure S4 in Supplementary Material at the late stage of *C. difficile* infection (CDI). The upper clump represents the pathogen core pathways, and the lower clump signifies the host core pathways at the late stage of CDI. The gray solid lines denote the protein–protein interaction; the red arrow lines are transcriptional regulation; the green dot lines signify protein translation; the gray blue dash lines represent protein secretion; and the purple clump with arrow and dash lines indicate the activity of reactive oxygen species (ROS). The abundant activities of CD0663 and the acetylation of fibronectin 1 (FN1) result in enhanced ROS production and a strong inflammatory response, while these counter mechanisms in turn increase the cellular stress of host cells. The endoplasmic reticulum (ER) stress response reflects that the accumulated cellular stress could risk the host cell. Therefore, the tissue damage caused by severe inflammation and the accumulated cellular stress eventually triggers the apoptosis process of host cells. In addition, *C. difficile* utilize DNA damage response and antioxidative proteins against human-produced ROS, and reduce the toxins production and cell growth rate for sporulation to transform to endospore.

The cross talk between host and pathogen has been extensively investigated. However, the epigenetic modulation and interspecies protein–protein interactions of CDI are still largely unknown. To further investigate the offensive and defense mechanisms between the host and pathogen, we rearranged the HPNs in Figures S3 and S4 in Supplementary Material from the perspective of signal transduction pathways. The rearranged core signal transduction pathways of HPNs in Figures S3 and S4 in Supplementary Material at the early and late stage of CDI are shown in Figures 3 and 4, respectively.

For the validation of our identified host–pathogen protein– protein interactions in Figures 3 and 4, we surveyed the existing literature for studies reporting recognized host–pathogen interactions during CDI. The interaction [CD2787, fibronectin 1 (FN1)] in both Figures 3 and 4 has been experimentally verified (11). CD2787 is involved in cell adhesion by binding to FN1, and degrading host extracellular matrix proteins. Meanwhile, the interaction (CD0660, HSP90B1) in Figure 3 and (CD0660, HSP90B2P), (CD0663, HSP90B1), and (CD0663, HSP90B2P) in Figure 4 can be verified by Chaves-Olarte et al. (12) and Na et al. (13), which revealed that *C. difficile* toxins can enter host cells through GP96. Similarly, the suggested cytotoxic interactions (CD0660, CDC42), (CD0663, CDC42), (CD0660, RHOA), and (CD0663, RHOA) in Figure 3, and (CD0663, RAC1) in Figure 4 can also be verified in Just et al. (4, 14) and Chen et al. (15).

While in the genetic-and-epigenetic core pathways at the late stage of CDI shown in Figure 4, a new pathogen protein CD1466 was secreted out by *C. difficile*. CD1466 encoding an ATP-binding protein belongs to the ABC-type transport system, which is the same as CD0478 in the early stage. The results show that an ATP-binding cassette transporter CD1466 interacts with host receptors SNW1 and EGFR, causing various signal cascades in the host cell. The interaction between ATP-binding cassette transporters and SNW1 has been reported by mass spectrometry (16). In addition, the suggested interaction between CD1466 and EGFR during CDI could provide *C. difficile* with a fast and efficient way to change transporter function (17). SNW1 in Figure 4 was observed to enter the cytoplasm and then interact with TFs *via* acetylation to induce immunity-related processes. In addition, EGFR is participating in several cellular responses, including GTPase activity, immune response, inflammation, and apoptosis. In Figure 4, the downstream TFs of EGFR-mediated pathways include NFIC and NFKB1, involved in inflammatory response. The inflammatory response triggered by CD1466 could be used by host cells to remove invasive pathogens. However, severe inflammation could also induce tissue damage of host cells. The tradeoff of inflammation in CDI will be discussed in later sections. In addition to CD1466, CD0663 and CD0660 are also secreted in the late stage of CDI (Figure 4). The result suggests that CD0663 can bind to CD46, HSP90B1, HSP90B2P, and RAC1 (Figure 4). The suggested interaction between CD0663 and RAC1 in the result (Figure 4) can be supported by the previous report (14). By mass spectrometry, the interaction between CD0663 and HSP90B1 has also been reported (18). Therefore, according to sequence-homology and the result in Figure 4, we suggested that the interspecies PPIs between pathogen CD0663 and host CD46 and HSP90B2P could occur during CDI.

### Drug Targets Prediction for Treating CDI

Considering the crucial role of CD2787 (cwp84) in pathogenesis at the early stage of CDI, we recommend CD2787 as a potential drug target in the prevention of CDI. Moreover, other cell-wall proteins participating in the infectious process, such as CD0440, CD0237, CD0266, and CD1987 are also potential drug targets since the inhibition of these cell surface proteins may reduce the occurrence of cell adhesion. In Figures 3 and 4, four pathogen genes (*CD0130*, *CD3256*, *CD1275*, and *CD2781*) were previously predicted as essential survival/growth genes by applying flux balance analysis (FBA) and synthetic accessibility (SA) to a curated *C. difficile* metabolic network, as well as transposon-directed insertion site sequencing (TraDIS) to the *C. difficile* transposon mutant library (19, 20). However, CD0130 and CD3256 have important human-homologs, MAT2A and VARS, respectively (19). The inhibition of these proteins may result in unpredictable dysfunction of host cells. Therefore, CD1275 and CD2781 are more conservative choices for further drug design. Similarly, proteins involved in the defense mechanisms employed by *C. difficile* against reactive oxygen species (ROS), including CD0141 and CD2115 in the early stage of CDI, and proteins responsible for redox-state homeostasis (CD1690 and CD1631) in the late stage of CDI, have human-homologs (19). Therefore, the three remaining anti-ROS enzymes (CD2356, CD0171, and CD0179) are recommended as potential targets to repress the defense mechanisms of *C. difficile*. The heavy economic burden of CDI results from its high recurrence rate, suggesting that the inhibition of spore formation of *C. difficile* is a feasible therapeutic way to reduce the recurrence rate of CDI. In our results, the key sporulation pathway members included CD1214, CD2629, and CD2643. These proteins could become targets of further drug design.

In addition to the inhibition of pathogen mechanisms, another strategy of drug design includes the promotion of host defense mechanisms. It has been reported that low doses (0.2 ng/ml) of CD0660 is sufficient to induce significant cell rounding *in vitro* (21), suggesting that amplification of host defense appear to be required. In our results, the major pathogenic effects induced by *C. difficile* pathogenic factors include tight junction/cytoskeleton breakdown and the accumulation of endoplasmic reticulum (ER) stress, which result from the inactivation of Rho GTPases (RHOA, CDC42, and RAC1) and the dysfunction of chaperone proteins (HSP90B1, HSPA5, and HSP90B2P), respectively. Therefore, we aim to increase the expression of these genes to maintain the activities of these dysfunction components. The severe inflammation and apoptosis process, induced by NFKB1, REL, and IL-8, are responsible for cell death at the late stage of CDI. Though the activities of immune response and inflammation are important counter mechanisms against bacteria, the overexpression of these related genes cause tissue damage and apoptosis of host cells. Therefore, we suggest repressing the expression of NFKB1, REL, and IL-8 to relieve these activities.

Our results show that CD2356, CD0171, and CD0179 participate in the defense mechanisms of *C. difficile* against oxidative stress. The co-operation among these proteins provides a well-designed protection against human-produced ROS. The inhibition of these antioxidative proteins facilitates the host eliminating ability against pathogens, and the scattered ROS can induce the rapid necrosis of pathogen cells. Therefore, we recommend that CD2356, CD0171, CD1064, and CD0179 are potential drug targets for further drug design (see supplementary methods and Table S2 in Supplementary Material and Figure S5 in Supplementary Material).

## Discussion and Conclusion

### Comparison of the Pathogen Core Network with Previously Proposed Essential Genes or Proteins in CDI

During the pathogenesis of the bacterium, essential genes are required for the survival and growth of the pathogen. The absence of essential genes results in a decreased growth rate or death of the organism. The pathogen core networks extracted from GEINs display not only essential proteins required for the survival of the pathogen but also important enzymes that contribute to the pathogenesis and defense mechanism of the pathogen against various stress conditions. Here, we compare HPNs with the existing predicted essential proteins of *C. difficile* to show the difference and advantage of HPNs.

Recently, by applying FBA and SA to a curated *C. difficile* metabolic network, Larocque et al. predicted 76 essential *C. difficile* genes (19). Seven *C. difficile* proteins (CD2664, CD2335, CD3550, CD0198, CD1225, CD0130, and CD0123) in the HPN of the early stage of CDI (Figure S3 in Supplementary Material) and three *C. difficile* proteins (CD2588, CD1816, and CD0130) in the HPN of the late stage of CDI (Figure S4 in Supplementary Material), encoded by *C. difficile* genes appear to be required for the survival of the pathogen, have been identified in this study. Moreover, applying transposon-directed insertion site sequencing (TraDIS) to the *C. difficile* transposon mutant library led to the identification of 404 genes with no transposon insertion in the library, which can be considered as essential genes for *C. difficile* growth (20). Thirteen *C. difficile* proteins (CD2664, CD2335, CD0067, CD3550, CD3540, CD0198, CD1255, CD2714, CD3256, CD1316, CD0095, CD2739, and CD0123) in the HPN of the early stage of CDI (Figure S3 in Supplementary Material) and 15 *C. difficile* proteins (CD3170, CD2588, CD2744, CD2771, CD2462, CD3304, CD2781, CD3540, CD1145, CD2461, CD2793, CD0059, CD1275, CD0052, and CD1767) in the HPN of the late stage have been identified in this study as the products of likely essential genes for the growth of *C. difficile*. Overall, 15 pathogen genes in the HPN of the early stage of CDI, and 17 pathogen genes in the HPN of the late stage, were previously identified as likely essential genes of *C. difficile* (Table S1 in Supplementary Material).

Besides these previously identified essential proteins, HPNs also provide numerous crucial *C. difficile* enzymes that participate in the offensive and defensive mechanism utilized by the pathogen, such as well-known toxins (CD0660 and CD0663), toxin-regulators (CD0659, CD0661, and CD0664), cell-wall proteins (CD2787, CD1987, CD0237, and CD0440) that provide cell-adhesion abilities, defensive proteins (CD0141, CD0171, CD2115, CD1631, and CD1690) against ROS, and sporulation-related proteins (CD1214, CD2643, CD2629, and CD1511). The presence of these proteins in HPNs reveals that there are various cross-talk activities between the host and pathogen in the offensive and defensive mechanism.

### The Induced Homeostatic, Apoptotic, Intestinal Inflammatory Responses in CDI Patients Mediated by Acetyltransferase and Methyltransferase Proteins

The interactions between the small GTPase (CDC42) and downstream effectors (GRB2 and KBTBD7) suggest that the dynamic of cytoskeleton homeostasis can be affected by *C. difficile* toxins and host epigenetic activities to influence the change of cell shape displayed in Table 3. Similarly, at the late stage of CDI (Figure S4 in Supplementary Material), the subunits (NFKB1 and REL) of the NF-κB complex are regulated by acetyltransferase proteins (GCNT2 and B3GNT6) and the methyltransferase protein (PRDM14), resulting in the assembly of the NF-κB complex and the induction of apoptosis in host cells. The change in the methylation level of HSPA5 has been proposed in Hesson et al. (22) *via* NOME-Seq analysis of HSPA5 in intestinal disease, suggesting that altered nucleosome positioning induces differences in accessibility. In addition, in the late-stage HPN shown in Figure S4 in Supplementary Material, the significant difference in the basal level of the NF-κB subunit NFKB1 gene reflects the change of methylation level, which is consistent with a previous study showing that NFκB1 is hypomethylated in intestinal inflammation (23). These identified HPNs reveal the ability of acetylation and DNA methylation likely to alter cellular functions in order to adapt to bacterium infection.

### Cross-talk Mechanisms in CDI Patients Mediated by Ubiquitin and Acetyltransferase Proteins

Once the bacterium is attached to the surface of Caco-2 cells in the early stage of infection (Figure 3), the cell surface-associated cysteine protease CD2787 (cwp84) of bacterium interacts with FN1 on the host membrane. This negative interaction (CD2787, FN1) and the MIB2-induced ubiquitination of FN1 suggest that CD2787 could provide a degrading activity on FN1, which can be supported by the biological experiment (11). Since FN1 is involved in the maintenance of cell shape, it can bind cell surface and various compounds, including actin. We suggested that the degradation of FN1 results in the morphological change of Caco-2 cell or the disturbance of the actin cytoskeleton. The results also suggest that three pathogen proteins (CD0660, CD0663, and CD0478) secreted by *C. difficile* interact with four host receptors [FPR1, SCARA3, HSP90B1, and Arrestin Beta 2 (ARRB2)]. FPR1 has been reported as the receptor of CD0660 (TcdB) (24). Owing to its interaction with toxins (CD0660 and CD0663) and ubiquitination modified by ARIH2, we suggested that *C. difficile* toxins enter the host-cell cytoplasm *via* receptor-mediated endocytosis. For *C. difficile* toxins, another identified human cell surface receptor HSP90B1, which encodes a member of HSP 90 kDa family, plays a role in protein folding. It has been reported that the ER chaperones HSP90B1 and HSPA5 could be significantly downregulated by HDAC inhibition at the protein level (25). HSP90B1 has been identified as a cell surface receptor for *C. difficile* proteins, but HSP90B1 did not respond to CD0660 (13). The observation can be supported by the result in the GEIN that HDAC inhibition impairs the binding affinity of HSP90B1 for CD0660.

The interaction between CD0663 and CD46 (Figure 4) indicates that pathogen CD0663 could be the extracellular stimuli of CD46, and potentially responsible for CDI to recruit phagocytic cells such as neutrophils to clear toxins and whole microbes in the early stage. The result in Figure 4 suggests acetylation and interaction with toxins (CD0660 and CD0663) of HSP90B2P, which participate in impairing its chaperone-ability. Interestingly, we noticed that there are more host proteins influenced by CD0663 than CD0660 in the late stage of infection. By contrast, CD0660 binds more host proteins than CD0663 in the early stage (Figure 3). These results provide an explanation for the perennial argument about the cytotoxic responsibility of CD0660 (TcdB) and CD0663 (TcdA), suggesting that both toxins are likely essential for pathogenesis. The results suggested that this role change may result from CD2973-induced acetylation of CD0663 in the late stage (Figure 4). The rise of CD0663 activity could be the turning point for the progression of CDI. We observed that another host protein, FN1, could also change its role from the early stage to the late stage of CDI. FN1 is repressed by ubiquitination and CD2787-induced degradation in the early stage of CDI (Figure 3), but the CD46-triggered activation and NAT8L-induced acetylation of FN1 increased the corresponding expression levels (*p*-value < 8 × 10^−3^) despite the influence of CD2787, thus allowing FN1 to trigger downstream activities in the late stage of CDI in Figure 4. Therefore, we suggested that CD0660 functions prior to CD0663 and triggers rapid responses in the early stage of infection and CD0663 works actively in the late stage.

**Figure 5:**
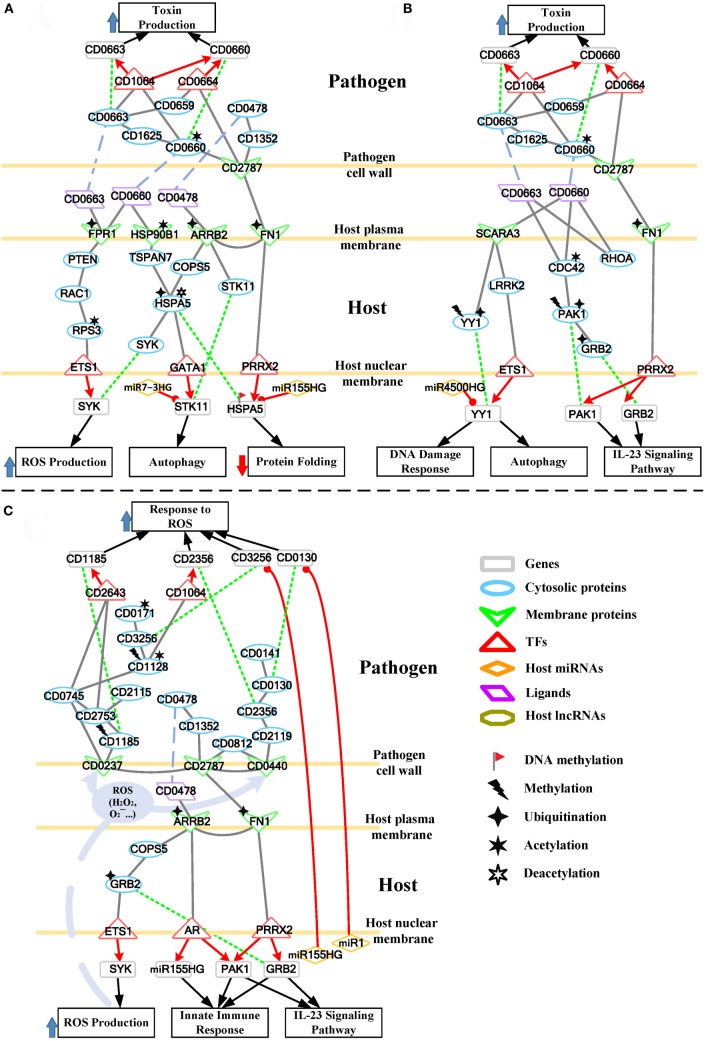
The host/pathogen cross-talk mechanism through core pathways at the early stage of *C. difficile* infection (CDI). **(A)** The secreted pathogenic factors of *Clostridium difficile* (*C. difficile*) and their induced cytopathic effect in Caco-2 cells; **(B)** the remedial schemes employed by host cells in response to *C. difficile* toxins; **(C)** offensive mechanisms *via* host-secreted reactive oxygen species (ROS), microRNA (miRNAs), and immune response, and thus the defense mechanism of *C. difficile* at the early stage of CDI. The red arrow lines denote transcriptional regulation; the gray solid lines indicate the protein–protein interaction; the green dot lines signify protein translation; the gray blue dash lines indicate protein secretion; and the purple clump with arrow and dash lines represent the activity of ROS. In **(A)**, *C. difficile* toxins hijack a RAC1-related pathway to promote ROS production in host cells. The significant change of acetylation level of heat shock proteins (HSP90B1 and HSPA5) and their interactions with toxins impair their chaperone-activity, resulting in the formation of misfolded protein and the induction of autophagy. As the remedial schemes for these injuries, in **(B)**, Caco-2 cells employ the DNA damage response and autophagy to deplete the damage caused by pathogens, and innate immune response to remove the invasive bacterium. The alternative routes of these counter mechanisms and the miRNA silencing used by host cells to interfere with the activities of pathogen are also displayed in **(C)**. Furthermore, the scheme in **(C)** shows the multiple pathways of *C. difficile* against the oxidative stress presented by Caco-2 cells.

**Figure 6:**
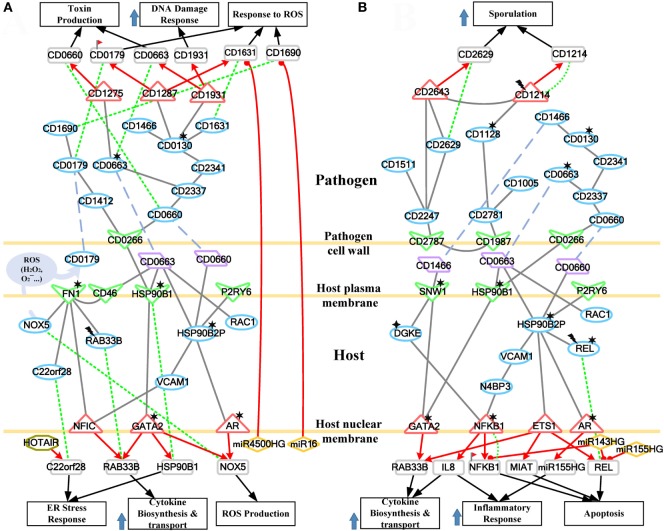
The host/pathogen cross-talk mechanism through core pathways at the late stage of *C. difficile* infection. **(A)** The enhanced reactive oxygen species (ROS) production and cellular stress of host cells, and the anti-ROS mechanism of *Clostridium difficile* (*C. difficile*); **(B)** the stress-induced apoptosis of Caco-2 cells and the leaving of *C. difficile*. The red arrow lines denote transcriptional regulation; the gray solid lines indicate the protein–protein interaction; the green dot lines signify protein translation; the gray blue dash lines indicate protein secretion; and the purple clump with arrow and dash lines represent the activity of ROS. The definitions of node symbols are the same as those defined in Figure 5. In **(A)**, the abundant activity of CD0663 and the acetylation of fibronectin 1 increase the production of ROS and trigger the formation and transport of cytokine, inducing a strong inflammatory response. However, the presence of endoplasmic reticulum (ER) stress response reflects that the accumulated cellular stresses also risk the survival of the host cell. Meanwhile, *C. difficile* employ DNA damage response and antioxidative proteins to counteract the oxidative stress. In **(B)**, the severe inflammation, accumulated oxidative stress and ER stress, and the activity of NF-κB complex trigger the apoptosis of Caco-2 cells. In addition, *C. difficile* actively transform to endospore to lie dormant and avoid the risking environment.

To further understand this, we separate the rearranged core pathways in Figures 3 and 4 based on different cellular responses and their corresponding pathways for further discussion. For the early stage of CDI, the genetic-and-epigenetic scheme of the core pathways (Figure 3) can be separated into three parts: the offensive mechanism of the pathogen and the corresponding pathogenesis of host cells (Figure 5A); the remedial actions employed by host cells in response to pathogen-induced injuries (Figure 5B); and the counter mechanisms of Caco-2 cells and the defense mechanisms of *C. difficile* (Figure 5C). During the late stage of CDI, the core pathways (shown in Figure 4) can be separated into two parts: the strong cellular responses triggered by epigenetic acetylation in host cells for depleting the tenacious pathogen at the late stage of CDI (Figure 6A) and the severe inflammation and apoptosis processes of Caco-2 cells as well as the endospore formation of *C. difficile* (Figure 6B).

### A Precise View of Pathogenic Effects and Host Responses at the Early Stage of *C. difficile* Invasion

#### Pathogenic Factors Utilized by *C. difficile* and the Resulting Pathogenesis in Caco-2 Cells

During CDI, the first event that initiates pathogenesis is cell adhesion of the two organisms. We identified that CD2787 plays an important role in cell adhesion by binding FN1 (Figure 5A), resulting in the degradation of the extracellular matrix proteins of host cells (11). On the pathogen side, CD2787 also interacts with the cytotoxin CD0660 (TcdB) to promote the secretion of toxins, and signals *via* the toxin regulator CD0664 (TcdC) to enhance the production of CD0660 (Figure 5A). On the host side, the enterotoxin CD0663 (TcdA) and cytotoxin CD0660 (TcdB) are the two major *C. difficile* pathogenic factors and the main causes of clinical symptoms of CDI. In Figure 5A, the result suggests that CD0660 can activate CD0663 *via* CD1625, a histidine kinase. Another route for CD0660 to control toxin production is through interaction with the pathogen transcriptional regulator CD1064 (Figure 5A). This protein regulates a wide range of genes, including *CD0660* and *CD0663* (Figure 5A). Similarly, CD0663 (TcdA) can enhance toxin production *via* CD1064 and CD0664 (Figure 5A). It should be noted that the toxin activator, CD0659, regulates CD0664 (Figure 5A). Therefore, the results suggest that higher expressions of CD0660 and CD0663 in the early stage of CDI (CD0660: *p*-value <5 × 10^−3^, CD0663: *p*-value <4 × 10^−3^) reflects the higher activities of toxins at this stage. Moreover, the result also suggested that CD2787 could activate an ATP-binding cassette transporter CD0478 through histidine kinase CD1352. CD0478 is involved in the antibiotic resistance of *C. difficile* (26). On the host side, we suggested that this protein, which interacts with human cell receptors, is responsible for the subsequent response of human cells in CDI, suggesting its potential immunogenicity.

A G protein-coupled receptor FPR1, which interacts with *C. difficile* toxins (Figure 5A), is involved in the activation of immune-related phagocytic cells. On the host side, the result in Figure 5A suggests that FPR1 can negatively interact with its downstream protein PTEN, a tumor suppressor protein. PTEN inhibition has been reported to activate the small GTPase RAC1 (27), which leads to the decreased PTEN expression (*p*-value <3 × 10^−3^) and the increased expression of RAC1 (*p*-value <2 × 10^−3^) during CDI infection. The activated RAC1 is characterized by its participation in assembly of the NADPH complex and, thus, the production of ROS in CD0660-infected Caco-2 cells (28). Interestingly, the results show that another glycosylation-independent pathway exists for RAC1 regulation of ROS production. The result shows that RAC1 activation of RPS3 mediates ETS1 translocation to the nucleus for transcription (Figure 5A). The SYK gene is regulated by ETS1 and is also involved in ROS production. The highly expressed SYK in the early stage of CDI (*p*-value <3 × 10^−3^) reflects an induced oxidative stress caused by ROS. Oxidative stress reflects the imbalance of the redox-state in a biological system. The overproduction of ROS or insufficient reducing agents in the system could lead to oxidative stress. Oxidative stress is usually activated by the host to kill pathogen. However, the simultaneous production of ROS and free radicals causes severe damage of cellular components, including lipids, proteins, and DNA. In the case of CDI, toxins hijack the RAC1-dependent pathway to generate ROS, resulting in oxidative-stress-mediated necrosis of host cells.

Heat shock proteins are usually activated to assist with the folding and refolding of cellular proteins in response to environmental stress. The result suggests that the deacetylation of HSPA5 can switch its folding ability into a degrading ability (29). Moreover, UBE2D3-induced ubiquitination of HSPA5 results in its repression in the early stage of CDI (*p*-value <2 × 10^−3^). The result shows that *HSPA5* gene is also inhibited by the activated miR155HG and its DNA methylation in the early stage of CDI. Therefore, the result suggests that the chaperone-ability and degrading activity of HSPA5 are repressed in the early stage of CDI.

Furthermore, the result suggests that the deacetylation of HSPA5 can activate the expression of Serine/Threonine Kinase 11 (STK11) *via* GATA1. STK11 is responsible for autophagy activation in response to the aggregation of misfolded proteins. However, the activated miR7-3HG directly inhibits STK11, which results in dysregulated autophagy activation.

#### Caco-2 Cells Adopt Autophagy, DNA Damage Response, and the Activation of PAK1 and GRB2 As Remedial Schemes in Response to Pathogen-Induced Damage

SCARA3 and LRRK2, which encode a ROS scavenger protein and a leucine-rich repeat kinase, respectively, are employed to counteract environmental stresses, including overproduction and secretion of ROS, and to be responsible for induce autophagy initiation, respectively. The result suggests that LRRK2 can enhance the expression of YY1 by repressing ETS1 (Figure 5B). The activated LRRK2 and YY1 (*p*-value <2 × 10^−3^ and <1 × 10^−3^, respectively) in the early stage of CDI also support this relationship. These identified regulatory interactions suggest that host cells recruit numerous proteins and genes to maintain the redox-state balance and protein homeostasis.

Over the past decade, *C. difficile* toxins (CD0660 and CD0663) have been characterized by their capacity for glycosyltransferase-dependent inactivation of host Rho family GTPases (RHOA, CDC42, and RAC1). It has been shown that these modifications result in cytoskeleton rearrangement, severe inflammation, and subsequent apoptosis in human cells (3). In Figure 5B, the result suggests that CDC42, which interacts with CD0660 and CD0663 and is modified by acetylation, impairs its association to the CDC42-activated kinase 1 (PAK1) and subsequent GRB2. The inhibited PAK1 and GRB2 (PAK1: *p*-value <2 × 10^−3^, GRB2: *p*-value <4 × 10^−3^) also support this relationship. PAK1 and GRB2 are involved in activation of the immune response such as the interleukin-23 signaling pathway, which contributes to the regulations of cell adhesion, inflammation, and apoptosis in intestinal epithelial cells (30). Interestingly, the result suggests that PRRX2 functions as a double-edge sword (Figure 5B). The repression of PRRX2, resulting from CD2787-mediated degradation of FN1, not only decreases the efficiency of chaperone functions as previously mentioned but also unlocks the repression of *PAK1* and *GRB2* genes. This process can restore cytoskeleton homeostasis and initiate the interleukin-23 signaling pathway.

#### The Offensive Mechanisms of Caco-2 Cells and the Defense Mechanisms of *C. difficile* at the Early Stage of CDI

The evolutionary war between microbes and humans has lasted for thousands of years. In addition to the defense mechanisms developed against microbial infection, host cells also evolved strategies to kill pathogens. However, lipids and proteins from human cells cannot easily pass through bacterium cell walls and membranes. These offensive interactions exist but still remain largely unknown and generally species specific. The major means employed by host cells include the production of tiny and toxic molecules (ROS), recruitment of phagocytes (neutrophil, macrophages), and newly detected miRNA regulation (7). The production of ROS and immune response initiation has been discussed in previous sections. The alternative routes of these offensive processes are also shown in Figure 5C.

Through its interaction with FN1 and CD0478 (Figure 5C), ARRB2 can signal through the COP9 signalosome subunit COPS5 to positively activate GRB2. GRB2 can participate in cytoskeleton homeostasis and the innate immune response. The result in Figure 5C shows GRB2 activates ETS1 to upregulate *SYK* expression and, thus, ROS production. The co-operation of ROS and innate immune processes form an offensive mechanism to deplete pathogens. ARRB2 can also transmit stimulation signals to androgen receptor (AR) (Figure 5C). Once stimulated, AR dissociates from accessory proteins, translocates to the nucleus, and then induces the transcription of *PAK1* and *miR155HG* (Figure 5C). Both PAK1 and miR155HG are involved in the innate immune response. The feature of this pathway is the activation of miR155HG (Figure 5C). The high expression level of this miRNA increases the probability of passing through pathogen cell barriers (cell walls and cell membranes) to silence the pathogen gene *CD3256*. The relationship between host miRNAs and pathogen genes has recently been reported (7). This seminal observation revealed that the host can regulate and shape gut microbiota through miRNAs. The result suggests that miR155HG and miR1 can silence *CD3256* and *CD0130*, respectively (Figure 5C). CD3256 and CD0130 function in pathogen pathways that counteract host-produced ROS. This indicates that host cells can interfere with pathogen defense mechanisms and enhance the host’s ability to eliminate *C. difficile*.

Oxidative stress is usually employed by host cells to kill pathogens, but *C. difficile* toxins enhance ROS production through a RAC1-mediated pathway, causing injury to the host and DNA damage, to avoid or defend against ROS (Figure 5A). When ROS is released at the interface between the host and pathogen, pathogen cell-wall proteins are the first to be encountered in CDI. In Figure 5C, on the pathogen side, the result suggests that the cell-wall proteins CD2787 (cwp84) and CD0440 (cwp27) can positively activate CD0812, which is a universal stress protein. Universal stress proteins can promote endurance under stress conditions, such as heat, nutrient starvation, chemical agents, and oxidative stress. The homoserine kinase CD2119, which participates in several essential metabolic pathways, is also employed by CD0440 to activate CD2356 (Figure 5C). CD2356 is a thioredoxin reductase that can remove superoxide radicals and balance the redox state of pathogens. A high expression level of CD2356 in this stage (*p*-value <6 × 10^−4^) could function to resolve oxidative stress. In addition to performing its own activity, the result also suggests that CD2356 can urge CD0130 to interact with CD0141 (Figure 5C). CD0130 encodes an S-adenosylmethionine synthase and has been identified as an essential survival gene of *C. difficile*, and the absence of this gene will result in gaps in metabolic networks and biomass decrease (19). Its metal ion binding ability can help CD0141 with exporting copper ions. Copper (Cu) is an essential element for most species, including bacteria. However, overexposure to Cu is toxic. In fact, copper and its alloys are natural antimicrobial materials that have long been used as bactericides before antibiotics were discovered. The toxicity of copper ion is primarily due to its reaction with human-produced H_2_O_2_ (a member of ROS) to generate the hydroxyl radical (⋅OH), which damages pathogen lipids, proteins, and DNA. To reduce this risk, the copper homeostasis protein CD0141 is activated to export copper from pathogen (Figure 5C). Interestingly, we identified that *CD0130* is silenced by the host miRNA miR1 (Figure 5C). This regulation reduces the probability of CD0130-CD0141 co-operation, thus interfering with pathogen defense mechanisms. Unfortunately, the expression of CD0130 remains high in the early stage of CDI (*p*-value <3 × 10^−3^), and this may be due to the lack of miR1 upregulation.

CD0237 (FliD) encodes a flagellar protein that together with CD2787 participates in *C. difficile* adhesion. In Figure 5C, the result suggests that this cell surface protein responds to ROS by activating CD1185 and CD2753. CD1185 is a diguanylate kinase protein that participates in the formation of c-di-GMP, a ubiquitous second messenger involved in bacteria biofilm formation and pathogen aggregation (31). The aggregated pathogen cells also promote the formation of biofilm. CD2753 is also a c-di-GMP-signaling component that is specific to *C. difficile* 630. The assembly of these second messenger subunits induces various cellular responses. One downstream TF, CD2643, which regulates the sporulation process, is activated to transcriptionally regulate *CD1185*, creating a self-activation loop. CD1185 is methylated, but its high expression (*p*-value <2 × 10^−3^) at the early stage and the establishment of the feedback loop reveals the high activity of c-di-GMP and, thus, the formation of biofilm, which is a powerful scheme against stress conditions, including oxidative stress. CD2115, a copper-transporting ATPase, is activated by CD2753 (Figure 5C), playing a similar role as CD0141. The existence of similar pathways modulating copper homeostasis indicates the important role of Cu ion in oxidative stress by highlighting the redundancy employed by pathogens to counteract oxidative stress.

Furthermore, in Figure 5C, the result suggests that CD0237 (FliD) can transmit signals *via* its downstream flagellum subunit CD0745, a putative chemotaxis protein, to trigger CD1128-mediated DNA replication. CD1128 is the DNA polymerase 1 (PolA) of *C. difficile*, and the initiation of DNA replication triggers bacterial reproduction. The increased amount of pathogen promotes the concentration of toxins and the resistance against stress conditions. CD1128 is methylated by CD2726 (Figure 5C), but its high expression level (*p*-value <5 × 10^−3^) and the positive interaction with CD0745 suggest that pathogen still recruits this protein for DNA replication. CD1128 then positively activates the transcriptional regulator CD1064 to regulate *CD2356* (Figure 5C). Another CD1128-mediated anti-ROS pathway includes CD3256 and CD0171. CD3256 (valS) is a valine-tRNA ligase and has been predicted to be an essential gene for the growth of *C. difficile* (20). Its activation ability to CD0171 is important for pathogen since CD0171 encodes a key redox-sensing regulator. CD0171 is only active as a repressor when the intracellular NADH/NAD^+^ ratio is low, thus regulating the redox-state of the pathogen (32). Interestingly, the result suggests that the host could interfere with this antioxidative defense process through miRNA silencing (Figure 5C). miR155HG is upregulated in the host cell (*p*-value <4 × 10^−3^) and identified as a repressor of CD3256. The inhibition of CD3256 by miR155HG results in not only redox-state disturbance but also pathogen-biomass decrease since CD3256 was predicted as an essential component for the growth of *C. difficile* (20).

### The Strong Cellular Activities of Caco-2 Cells and the Infection Results of Host and Pathogen at the Late Stage of Infection

#### The Emphasized ROS Production and Stress-Accumulation of Host Cells, and the Failure of Antioxidative Defense Mechanisms in *C. difficile*

When CDI proceeds to the late stage, pathogen toxins exist in host cells, and the bacteria continue to secrete pathogenic factors into the interface between the two species. In this situation, the scattered CD0663 binds the host membrane protein CD46 (Figure 6A), triggering various cellular responses and the complement system. As discussed above, CD2973-induced acetylation of CD0663 could promote its activity in the late stage. This in turn increases the probability of CD0663–CD46 interaction and the following responses. CD46 activates these processes through FN1 (Figure 6A). For example, FN1 can trigger ROS production by activating NOX5. NOX5 is a key member of the NADPH oxidase complex and is also responsible for superoxide generation. Unlike the indirect transcriptional regulation utilized in the early stage, ROS production *via* NOX5 in the late stage provides a much rapid and efficient antimicrobial effect since it is directly activated by host cell surface FN1 and immediately released into the interface (Figure 6A). RAB33B encodes a small GTP-binding protein and plays a role in vesicular transport in protein secretion, such as cytokine release. RAB33B is repressed by methylation (Figure 6A), but its high expression level (*p*-value <1 × 10^−3^) and positive interaction with FN1 indicate that host cells enhance protein transport through RAB33B (Figure 6A). In addition to direct interaction, FN1 can transmit signals through the nuclear factor NFIC to upregulate the expression of *RAB33B* (Figure 6A).

Another important downstream protein of FN1 is C22orf28 (Figure 6A). Referred to as RTCB, C22orf28 was recently reported as an essential component in the ER stress response (33). The ER stress response is a cellular stress response resulting from the accumulation of unfolded proteins in the ER. The major aim of the ER stress response is to refold or degrade misfolded proteins, thus relieving the ER stress. If this objective cannot be achieved in a certain time span, cells then activate apoptosis. The presence of C22orf28 reveals that the prolonged accumulation of misfolded proteins from the early stage induces ER stress and risks the survival of host cells. To relieve the stress, host cells employ lncRNA HOTAIR to activate *C22orf28* (Figure 6A), but the decreased level of HOTAIR (*p*-value <3 × 10^−3^) may in turn limit the activity of C22orf28. Overall, FN1 can be activated by CD46 and modified by NAT8L-triggered acetylation, which enhance the expression of FN1 and, therefore, promote cellular processes such as ROS production, ER stress response, and vesicular transport (Figure 6A). Therefore, the result suggested the presence of CD46 and the acetylation of FN1 as critical events during the progression from the early stage to the late stage of CDI.

Similar to the early stage, the chaperone function of HSP90B1 is impaired by the acetylation and interaction with *C. difficile* toxin CD0663 (Figure 6A), which results in misfolded protein formation. This result suggests that HSP90B1 is a conserved receptor for *C. difficile* toxins. To improve the impaired chaperone-ability, HSP90B1 urges the TF GATA2 to regulate its own expression. The acetylation of GATA2 also enhances its transcription ability to regulate the transcription of both *NOX5* to improve ROS production and *RAB33B* to strengthen cytokine release (Figure 6A).

In addition to HSP90B1, another HSP 90 kDa member, HSP90B2P, is influenced by *C. difficile* toxins (Figure 6A). *C. difficile* toxins (CD0663 and CD0660) can directly interact with HSP90B2P to repress the protein-folding ability of HSP90B2P in the host cells (Figure 6A). The acetylation of HSP90B2P also impairs its own chaperone function. In that case, HSP90B2P shows a similar response as HSP90B1. It can upregulate the expression of *NOX5* through AR, whose acetylation could promote its regulatory ability. HSP90B2P also activates the nuclear factor NFIC *via* the signal transduction protein VCAM1 (Figure 6A). Activated NFIC then upregulates the expression of *RAB33B* (Figure 6A). These same responses presented by HSP90B1 and HSP90B2P reveal that *C. difficile* toxins, whether intra- or extracellular, could affect the function of HSP90 proteins in a similar manner. The third manner in which toxins affect HSP90 proteins is through the inactivation of RAC1 (Figure 6A). The toxin-induced glycosylation of RAC1 leads to the production of UDP, a putative “danger signal” and the ligand for the receptor P2RY6 (34). The secreted UDP can alarm neighbor cells by binding to P2RY6 and, therefore, activate HSP90B2P-related processes (Figure 6A).

Host miRNAs, as mentioned above, can pass through pathogen cell walls and membranes, enter the cytosol, and then silence pathogenic genes. In the late stage of infection, the result suggests that host miRNAs, miR4500HG, and miR16, can inhibit the pathogen genes *CD1631* and *CD1690*, respectively (Figure 6A). Both silenced genes encode protective proteins against oxidative stress (CD1631: superoxide dismutase, CD1690: thioredoxin). This result is similar to the previous stage, suggesting that host miRNAs may target anti-ROS genes in *C. difficile*.

In response to the enhanced ROS production and the activities of the complement system, *C. difficile* performs multiple methods to counteract host mechanisms. In Figure 6A, the flagellar sigma factor CD0266 (FliA) replaces CD2787 to control toxin production and induce defense mechanisms against ROS. It can directly interact with CD0660 (TcdB) or activate another toxin, CD0663 (TcdA), *via* the transmembrane protein CD2337 (Figure 6A). The downstream target of CD0663 is a GTP-sensing transcriptional repressor CD1275 (CodY) (Figure 6A). This protein plays an important role in pathogen growth repression and its corresponding gene has been predicted as an essential gene for the growth of *C. difficile* (20). The presence of this protein indicates that pathogens transform from rapid growth to the stationary phase and reflect the risk situation presented by the host cell. Interestingly, the high expression of CD1275 (*p*-value <1 × 10^−3^) and the decreased expression of CD0660 (*p*-value <3 × 10^−3^) reveal that CD1275 in turn inhibits the expression of toxins, which is consistent with a previous study (35). CD2337 can also activate the metabolic dehydratase CD2341 and consequently its downstream essential protein CD0130 (Figure 6A). CD0130 plays a central role in this pathway to trigger various responses. However, a recent study indicated that the acetylation of the CD0130-homolog in *E. coli* impairs the corresponding enzymatic activity (36). The result in Figure 6A shows that CD0130 can activate the release of pathogenic factor CD1466 and signal *via* CD1931 (LexA) to regulate the toxin *CD0663*. CD1931 is a transcriptional repressor that can repress toxins and a number of genes involved in the DNA damage response (37). The impaired enzymatic activity of CD0130 results in low activity of CD1931 (*p*-value <1 × 10^−3^) and, thus, initiates the DNA damage response to remove ROS-induced DNA damage. The result suggests that CD0130 also employs alternative proteins to counteract ROS, such as superoxide dismutase CD1631, a conserved and powerful antioxidative protein, and CD1287, a fur family transcriptional regulator (Figure 6A). CD1287 can enhance the expression of *CD1631* and *CD0179* (Figure 6A), another defensive protein against H_2_O_2_, to eliminate ROS (38). However, these protective activities are limited by the acetylation of CD0130. Furthermore, DNA methylation of *CD0179* and host miR4500HG-induced silencing of *CD1631* (Figure 6A) also repress antioxidative defense mechanisms, resulting in DNA damage and component damage of the pathogen.

Since the function of CD1412 is currently unknown, the results suggest that it may participate in the defense mechanism triggered by CD0266 (Figure 6A). Once activated by CD0266, CD1412 then triggers CD0179 and the downstream CD1690 protein (Figure 6A). CD0179 encodes a glutamate dehydrogenase and is secreted by the pathogen, forming a protective layer against H_2_O_2_ (38). Its downstream target, thioredoxin CD1690, can co-operate with CD0179 to remove ROS around *C. difficile*. Activation of the CD1412-mediated pathway and the high expression of CD1287 indicate that *C. difficile* recruits these proteins against oxidative stress. However, the DNA methylation of *CD0179* and host miRNA-induced silencing of *CD1631* and *CD1690* confer limited efficiency.

#### The Apoptosis Process Triggered by Severe Inflammation, Accumulated Cellular Stresses of Caco-2 Cells, and the Leaving of *C. difficile via* Endospore Formation

Stimulated by CD1466, SNW1 participates in the NOTCH signaling pathway to modulate NF-κB activity. The result in Figure 6B suggests that SNW1 can enhance the transcription ability of GATA2 and, therefore, improve RAB33B-meidated protein secretion. In addition, SNW1 can activate NFKB1 through the diacylglycerol kinase (Figure 6B), which is involved in several immune-related pathways, including the interleukin-3, -5 signaling pathways. The activation of NFKB1 is an important event of CDI, and the acetylation of this NF-κB subunit could enhance its transcription ability to upregulate IL-8, a major cytokine for neutrophil recruitment (Figure 6B). Tight junction breakdown occurs at the early stage, allowing recruited neutrophils to enter the lumen and then clear pathogens by phagocytosis. However, neutrophil infiltration can also cause severe tissue damage in the host cell. In fact, neutrophil infiltration is an important clinical feature induced by CDI. Another important property of NF-κB is its ability to regulate apoptosis. In Figure 6B, the chaperone-ability of HSP90B2P is impaired by acetylation and the interaction with toxins. The prolonged ER and oxidative stress, initiated in the early stage, eventually trigger apoptosis *via* the signaling proteins VCAM1 and N4BP3 (Figure 6B). NFKB1 can upregulate its own expression and together with REL, another NF-κB subunit activated by HSP90B2P, promote NF-κB complex activity, thus initiating apoptosis. *NFKB1* and *REL* are methylated and silenced by miR143HG and miR155HG, respectively (Figure 6B). However, their high expression level (*p*-value <1 × 10^−3^) indicates that host cells undergo apoptosis in the final step of infection. Moreover, HSP90B2P utilizes ETS1 to enhance the expression of the lncRNA MIAT (Figure 6B), which has also been reported to participate in apoptosis in epithelial cells (39).

High levels of ROS and scattered neutrophils threaten the survival of *C. difficile*, forcing pathogens to leave the infection site. The decreased expression of the cell surface protein CD2787 (*p*-value <4 × 10^−3^) represses the adhesion ability of *C. difficile*. The result suggests that another cell-wall protein, CD1987, utilizes the transmembrane protein CD2781 to initiate the CD1128-related DNA replication and the following sporulation (Figure 6B). CD1128 encodes DNA polymerase 1 (PolA) of *C. difficile*, and the acetylation of this protein can affect replication timing. DNA replication in bacterium triggers either pathogen reproduction or sporulation. Endospores are special survival structures of some bacteria, including the *Clostridium* genus. They are highly resistant to ultraviolet radiation, heat, desiccation, chemical agents, and oxidative stress, allowing bacteria to lie dormant for even centuries. The result in Figure 6B also suggests that *C. difficile* can initiate the sporulation process *via* CD1214. CD1214 is the stage 0 sporulation protein that helps pathogens transform to an endospore. CD1214 is methylated during infection but is activated by CD1128 (Figure 6B) and, thus, upregulates its own expression to enhance its activity.

In addition, the result suggests that CD2787 triggers another sporulation pathway *via* CD2247 (Figure 6B). CD2247 encodes a serine peptidase, which plays a crucial role in *C. difficile* spore germination. CD2247 also controls the sporulation process (Figure 6B). For instance, it can activate the biosynthesis of spore coat protein CD1511 and improve the activity of CD2629, which is an ATPase required for spore coat formation and proper localization. CD2247 also activates a crucial sporulation regulator CD2643 (SigE) (40), and this sigma factor can upregulate the expression of *CD2629* (*p*-value < 8 × 10^−3^) and co-operate with CD1214. These activities suggest that pathogens actively transform to an endospore form in the late stage of infection.

### Pathogenic Model of CDI

**Figure 7:**
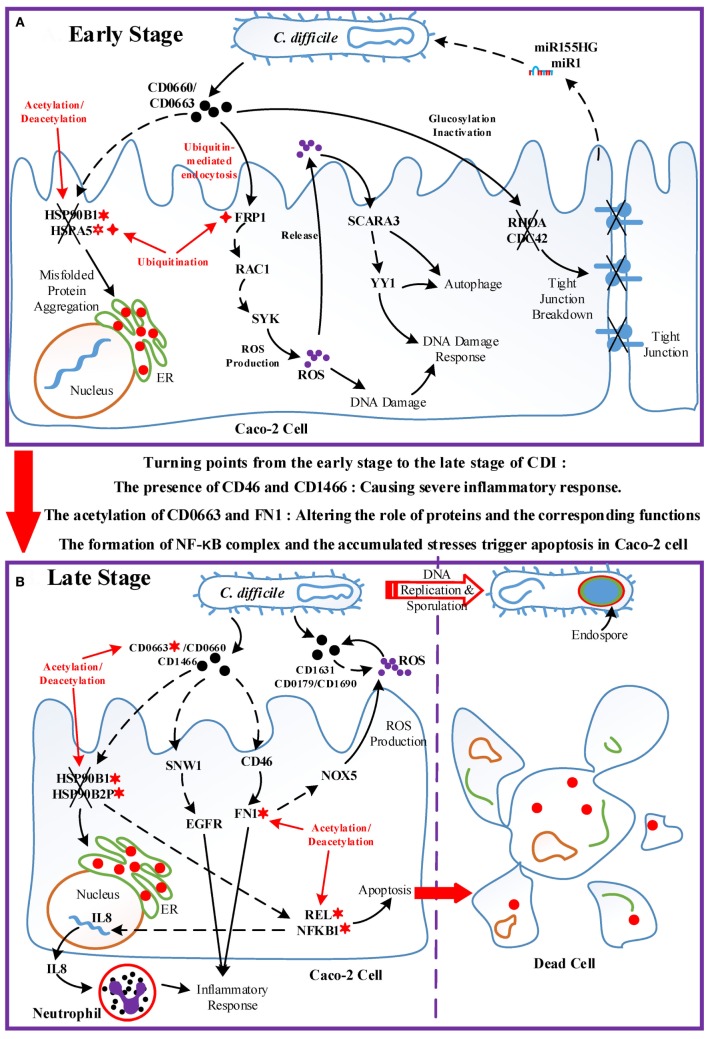
Overview of the pathogenic model of *C. difficile* infection (CDI). The upper panel (Figure 7A) displays the dominant role of epigenetic activity and the cross-talk interplay mechanisms between Caco-2 cells and *C. difficile* during the early stage of infection. The lower panel (Figure 7B) shows the molecular and epigenetic activities during the late stage of CDI and the outcome of both organisms. The cross marks signify the dysfunction or the breakdown of proteins and structures accordingly; the solid arrow lines represent the protein-interaction/cellular-response with literature support; the dash arrow lines denote the identified/predicted response; the red lines denote crucial epigenetic activities; the red dots in the endoplasmic reticulum of Caco-2 cell signify misfolded proteins; and the definitions of epigenetic activities are the same as those defined in Figure 5. In **(A)**, the epigenetic activities dominate the initialization of host responses toward bacterial invasion. The acetylation/deacetylation of heat shock proteins (HSPs) lead to the formation of misfolded proteins, the ubiquitination of FPR1 allows pathogen toxins enter Caco-2 cells *via* endocytosis and then trigger reactive oxygen species (ROS) overproduction, and the glucosylation of GTPases impair the homeostasis of cytoskeleton. In this situation, Caco-2 cells utilize DNA damage response and autophagy as defense mechanisms, and eliminate pathogens *via* ROS with the promotion of microRNA silencing. Between two stages, the acetylation of CD0663 and fibronectin 1, and the presence of CD46 and CD1466 serve as the turning points of the progression of infection, resulting in enhanced ROS production and severe inflammation as shown in **(B)**. The acetylation activity results in the dysfunction of HSPs and the assembly of NF-κB complex, inducing not only the production of IL8, which recruits neutrophils, but also the apoptosis process of host cells. To avoid the recruited phagocytic neutrophils and human-produced ROS, *C. difficile* cells activate the sporulation pathway to transform to endospore.

Using a systems biology approach to construct genome-wide GEINs *via* big data mining and two-sided genome-wide data identification, we investigated the pathogenic model of CDI (Figure 7). The result suggests that the acetylation of HSP90B1 and the deacetylation of HSPA5, and the interaction of these HSPs with pathogen toxins (CD0660 and CD0663), impair the chaperone-activity of host cell, resulting in the formation and accumulation of misfolded proteins. In addition, *C. difficile* toxins (CD0660 and CD0663) display their cytotoxicity by inactivating Rho GTPases (RHOA and CDC42) *via* glucosylation, causing the disturbance of actin cytoskeleton homeostasis and tight junction breakdown (3). Another central epigenetic activity is ubiquitination. The ubiquitination-mediated endocytosis of FPR1 allows *C. difficile* toxins enter host cells and then CD0660 and CD0663 hijack a RAC1-mediated pathway to activate SYK-dependent ROS production (24, 28). ROS production is a primary response of the immune system, but the toxin-activated overproduction of ROS and free radicals could damage host lipids, proteins, and DNA. To repair the damaged DNA, SCARA3 and YY1 are activated to initiate the DNA damage response. The dysfunction of HSPs together with YY1 trigger the autophagy process, removing misfolded proteins, and ROS-induced damage (41). As a countermeasure, host miRNA can pass through the pathogen cell wall and then repress pathogen genes, including the antioxidative proteins CD3256 and CD0130 to promote the eliminating ability of host cells.

When the infection proceeds to the late stage (Figure 7B), several events together result in the turning point of CDI. The result suggests that the acetylation of CD0663 promotes the activity of this toxin and, thus, increases the probability of CD0663 to bind to CD46. CD46 can activate the complement system and enhance ROS production *via* NOX5. The complement system guides neutrophils, which can be recruited by interleukin-8 to remove toxins and pathogens (42). In addition, FN1 can be activated by CD46 and modified by acetylation, and these interactions could enhance the activity of FN1 and its downstream inflammatory responses. Furthermore, the newly produced CD1466 displays its immunogenicity by interacting with SNW1 and EGFR, thus inducing cytokine production and inflammation. Taken together, the presence of CD1466 and CD46, and the acetylation of CD0663 and FN1, result in enhanced oxidative stress and a severe inflammatory response. Meanwhile, the acetylation of HSPs (HSP90B1 and HSP90B2P) and their interactions with toxins (CD0663 and CD0660) impair the chaperone-activity, aggravating the accumulated ER stress (43). These activities in turn increase the cellular stress of the host cell. For example, neutrophil infiltration and the increased NADPH oxidase could cause tissue damage of the host cell, and the presence of C22orf28 reflects high ER stress (33). On the other hand, these processes are assuredly life threatening to *C. difficile*. To counteract these threatening stresses, *C. difficile* utilize numerous redox-related proteins, including superoxide dismutase CD1631, extracellular glutamate dehydrogenase CD0179, and thioredoxin CD1690 in the defense against oxidative stress. Finally, the accumulated oxidative and ER stress trigger the apoptosis process *via* the NF-κB complex. For *C. difficile*, the high levels of ROS and scattered neutrophil risk the survival of pathogens. Therefore, *C. difficile* transform to endospores by activating the sporulation pathway, and then lie dormant for the next infection.

As discussed above, CDI is characterized by cytoskeleton dysfunction, severe inflammation, and subsequent apoptosis. The actin cytoskeleton breakdown is mainly induced by CD0663 and CD0660, but the correlation between cytoskeleton dysfunction and apoptosis remains unclear. Here, we suggest that the main cause of apoptosis during CDI is the accumulation of cellular stress, including oxidative and ER stress. The emergence of oxidative and ER stress has been reported in studies using CD0660-infection models (21, 44) but generates few attention and further researches. We suggested that Caco-2 cells activate the DNA damage response and autophagy to counteract these stressors in the early stage of CDI. Unfortunately, the accumulated stress and tissue damage caused by severe inflammation induce host cells to undergo apoptosis in the late stage of infection.

There has been a long-lasted argument about whether CD0660 or CD0663 is responsible for the cytotoxicity in host cells. Some early studies reported that only CD0660 is essential for the virulence of *C. difficile* and a number of patients with CDI are caused by CD0663-negative and CD0660-positive strains (45). However, a later study using toxin knockdown technology on *C. difficile* 630 indicated that both CD0660 and CD0663 are responsible for disease (46). Our identified results also support this conclusion. During the early stage of CDI, CD0660 functions prior to CD0663 and triggers numerous host responses, such as ROS production and chaperone dysfunction. In the late stage, acetylation enhances the activity of CD0663, which takes the place of CD0660, inducing a severe inflammatory response and aggravating ER stress. The CD0663-activating ability of CD0660 identified in our toxin-regulation pathways is also consistent with this temporal relationship. Without CD0660, CD0663 may be not sufficient to initiate pathogenesis in host cells in the early stage of CDI and the low expression level of CD0660 cannot trigger the subsequent apoptosis in the absence of CD0663 during the late stage of infection.

By contrast, unlike pathogenic effects on the host, the intraspecies interactions and cellular mechanisms inside *C. difficile* are largely unknown. We found that *C. difficile* could generate redox-balancing proteins against oxidative stress and utilize toxin production and bacterial reproduction as offensive mechanisms at the early stage of CDI. During the late stage of infection, the result suggests that *C. difficile* utilized anti-ROS proteins, including CD0179, CD1631, and CD1690, as well as the DNA damage response to counteract the oxidative stress presented by host cells. The decreased activities of toxin production and bacterium-cell growth, and endospore formation also reveal that *C. difficile* actively transform to endospores to leave the infection site. Finally, the molecular mechanisms of progression from the early stage to the late stage of CDI are investigated, and the potential drug targets are also proposed for further drug design.

The crucial events of progression in CDI, such as the acetylation of CD0663 and FN1, the chaperone dysfunction of HSPs, and the pathogen silencing induced by host miRNA, are mainly caused by epigenetic regulations identified in our systems biology method. These results suggest that epigenetic regulation plays an important role in the progression of infection since these cellular activities could change the cellular functions of host cells in a more rapid and efficient manner than the adjustment of gene regulation.

This study has three main limitations. (1) The reported results are modeling outcomes of two-sided genome-wide expression data and are speculations and not proven. (2) Several new potential interactions, especially interspecies PPIs, based on sequence-homology between *C. difficile* and *E. coli*, and the interspecies PPIs between *C. difficile* and *homo sapiens*, need for further experimental evidence. (3) The findings relate to only on strain of *C. difficile* and only once specific cell line, Caco-2 cells. The true complexity of an entire gut significantly complicates interactions with other cells/bacteria/host. This is great and valuable work but certainly has limitations. To the best of our knowledge, there are few studies focusing on the epigenetic modulation on pathogenic and offensive mechanisms in the host cell infected by *C. difficile*. In addition, no existing whole-epigenomic data of the host cells can be considered as the basis of such studies. We show that epigenetic regulation will play a more important role in host/pathogen cross-talk mechanisms in CDI, which provides a novel direction for deeper studies on molecular mechanisms for drug target predictions and multiple drug discovery. With the identification of GEINs and HPNs, our knowledge of the bioinformatics of *C. difficile* and the core proteins is bound to increase, driving further experimental hypotheses and investigation directions for cross-talk mechanisms, including the protein–protein interaction network (PPIN) and gene regulation network (GRN) between host and *C. difficile* during infection. These further studies will complementarily complete our genetic-and-epigenetic network, providing a novel basis for developing the whole-genome cellular network of CDI.

## Materials and Methods

### Overview of the Construction of GEINs and HPNs in Caco-2 Cells during the Early and Late Stage of CDI

A flowchart showing HPNs of the host and pathogen at the early and late stages of infection by big data mining, model construction, and network identification for investigating the cross-talk molecular mechanisms and inferring potential drug targets is shown in Figure 1. These processes can be divided into four steps: (1) big data mining and data preprocessing of host/pathogen gene/miRNA expression data (see supplementary methods); (2) construction of candidate GEIN, which consists of candidate host/pathogen intraspecies PPINs, candidate interspecies PPINs between host and pathogen, candidate host/pathogen gene/miRNA regulation networks, candidate miRNA regulation networks of host-miRNAs on host/pathogen-genes, and candidate lncRNAs regulation networks of host-lncRNAs on host-genes (see supplementary methods and Figure S6 in Supplementary Material); (3) identifying real GEINs of each stage from the candidate GEIN *via* system identification method and system order detection scheme, using the genome-wide microarray data of Caco-2 cells and *C. difficile* during infection; and (4) extraction of HPNs from the real GEINs using the PNP (see supplementary methods). We then investigated the crucial molecular mechanisms that contribute to the progression of CDI and inferred potential drug targets for further drug design.

### Genome-Wide Microarray Data of Caco-2 Cells and *C. difficile* during Infection

The two-sided microarray raw data proposed by Janvilisri et al. (6) has two parts. The first one contains the mRNA/miRNA expression profiles of three biological replicates of the Caco-2 cell line at 0, 30, 60, and 120 min postinfection with *C. difficile* 630. Each biological replicate contains two technical replicates (GEO accession number GSE18407). The Caco-2 cell line was cultured in Dulbecco’s modified Eagle’s medium at 37°C prior to infection. The second part contains the mRNA expression profiles of three biological replicates of *C. difficile* 630 in Caco-2 cells at 0, 30, 60, and 120 min postinfection (GEO accession number GSE18407; https://www.ncbi.nlm.nih.gov/geo/query/acc.cgi?acc=GSE18407). The platforms used in the host and pathogen were Phalanx Human OneArray and *C. difficile* 630/QCD32g58 array, respectively, which include 39,200 and 13,824 probes, respectively. The microarray data were validated using qRT-PCR. We took the average of the microarray data of the two technical replicates for further network identification. We applied one-way analysis of variance to calculate *p*-value of a gene using microarray data between early and late infection stages.

### Dynamic Models of GEINs for Caco-2 Cells and *C. difficile* during Infection

Since the candidate GEIN (see supplementary methods) was constructed by big data from numerous databases, experimental datasets, and literature, they contain some inevitable false-positive information. To address this, we built the dynamic model to characterize the molecular mechanisms of GEINs and to prune the false-positives in candidate GEIN, thus producing the real GEINs for Caco-2 cells and *C. difficile* during the infection process. For the PPIN of host proteins in candidate GEIN, the dynamic interaction model of the ith host protein can be described by the following dynamic equation:

(1) $$\begin{array}{l} p_{i}^{H}\left(t+1\right)=p_{i}^{H}\left(t\right)+\overset{F_{i}}{\underset{f=1}{\sum }}a_{\mathrm{if}}^{H}p_{i}^{H}\left(t\right)p_{f}^{H}\left(t\right)+\overset{Q_{i}}{\underset{q=1}{\sum }}c_{\mathrm{iq}}^{H}p_{i}^{H}\left(t\right)p_{q}^{P}\left(t\right)+\alpha_{i}^{H}g_{i}^{H}\left(t\right)\\ \;-\gamma_{i}^{H}p_{i}^{H}\left(t\right)+\kappa_{i}^{H}+\varpi_{i}^{H}\left(t\right),\text{ for }i=1,2,\ldots ,I,\alpha_{i}^{H}\geq 0\text{ and}-\gamma_{i}^{H}\leq 0 \end{array}$$

where $p_{i}^{H}\left(t\right)$, $p_{f}^{H}\left(t\right)$, $g_{i}^{H}\left(t\right)$ and $p_{q}^{P}\left(t\right)$ represent the expression levels of the *i*th host protein, the *f* th host protein, the *i*th host gene and the *q*th pathogen protein at time *t*, respectively; $a_{\mathrm{if}}^{H}$ and $c_{\mathrm{iq}}^{H}$ denote the interactive ability between the *i*th and *f* th host protein and between the *i*th host protein and *q*th pathogen protein, respectively; *F*_i_ and *Q*_i_ signify the number of host proteins and pathogen proteins that interact with the *i*th host protein; $\alpha_{i}^{H}$, $-\gamma_{i}^{H}$, and $\kappa_{i}^{H}$ indicate the translation rate from the corresponding mRNA, the degradation rate, and the basal level of the *i*th host protein, respectively. In general, the basal level $\kappa_{i}^{H}$ in Eq. 1 represents an unknown activity affecting the expression of the *i*th host protein other than those mentioned above, such as epigenetic acetylation and ubiquitination. $\varpi_{i}^{H}\left(t\right)$ denotes the stochastic noise of the *i*th host protein at time *t*. The biological meaning of Eq. 1 is that the expression level of the *i*th host protein can be affected by various molecular mechanisms including the host intraspecies PPIs $\sum_{f=1}^{F_{i}}a_{\mathrm{if}}^{H}p_{i}^{H}\left(t\right)p_{f}^{H}\left(t\right)$, interspecies PPIs $\sum_{q=1}^{Q_{i}}c_{\mathrm{iq}}^{H}p_{i}^{H}\left(t\right)p_{q}^{P}\left(t\right)$, protein translation $\alpha_{i}^{H}g_{i}^{H}\left(t\right)$, protein degradation $-\gamma_{i}^{H}p_{i}^{H}\left(t\right)$, basal level $\kappa_{i}^{H}$, and the corruption of stochastic noise $\varpi_{i}^{H}\left(t\right)$ In addition, the translation rate should be constrained to be non-negative and the protein degradation rate should be constrained to be non-positive in real PPIs.

For the GRN of host genes in the candidate GEIN, the dynamic model of the *j*th host gene can be described as follows:

(2) $$\begin{array}{l} g_{j}^{H}\left(t+1\right)=g_{j}^{H}\left(t\right)+\overset{I_{j}}{\underset{i=1}{\sum }}b_{\mathrm{ji}}^{H}p_{i}^{H}\left(t\right)+\overset{N_{j}}{\underset{n=1}{\sum }}e_{\mathrm{jn}}^{H}l_{n}^{H}\left(t\right)+\overset{I_{j}^{\prime }}{\underset{i^{\prime }=1}{\sum }}\overset{I_{j}^{\prime \prime }}{\underset{i^{\prime \prime }=1}{\sum }}x_{j\left(I_{j}^{\prime \prime }\left(i^{\prime }-1\right)+i^{\prime \prime }\right)}^{H}p_{i^{\prime }}^{H}\left(t\right)p_{i^{\prime \prime }}^{H}\left(t\right)\\ \;-\overset{K_{j}}{\underset{k=1}{\sum }}d_{\mathrm{jk}}^{H}g_{j}^{H}\left(t\right)m_{k}^{H}\left(t\right)-\lambda_{j}^{H}g_{j}^{H}\left(t\right)+\delta_{j}^{H}+\varepsilon_{j}^{H}\left(t\right),\\ \;\text{ for }j=1,2,\ldots ,J,-d_{\mathrm{jk}}^{H}\leq 0\text{ and }-\lambda_{j}^{H}\leq 0 \end{array}$$

where $g_{j}^{H}\left(t\right)$, $p_{i}^{H}\left(t\right)$, $m_{k}^{H}\left(t\right)$, and $l_{n}^{H}\left(t\right)$ indicate the expression levels of the *j*th host gene, the *i*th host TF, the *k*th host miRNA, and the *n*th host lncRNA at time *t*, respectively; $b_{\mathrm{ji}}^{H}$, $-d_{\mathrm{jk}}^{H}$ and $e_{\mathrm{jn}}^{H}$ represent the regulation ability of the *i*th host TF, the *k*th host miRNA, and the *n*th host lncRNA on the *j*th host gene, respectively; *I*_*j*_, *K_j_*, and *N_j_* denote the number of host TFs, host miRNAs and host lncRNAs, respectively, which regulate the expression level of the *j*th host gene; $p_{i^{\prime }}^{H}\left(t\right)p_{i^{\prime \prime }}^{H}\left(t\right)$ imply the *i*th host complex where $p_{i^{\prime }}^{H}\left(t\right)$ and $p_{i^{\prime \prime }}^{H}\left(t\right)$ represent the subunit 1 and 2 of the *i*th host complex, respectively; $I_{j}^{\prime }$ and $I_{j}^{\prime \prime }$ are the same, to denote the number of host complex, which regulate the *j*th host gene in the candidate GRN; $x_{j\left(I_{j}^{\prime \prime }\left(i^{\prime }-1\right)+i^{\prime \prime }\right)}^{H}$ signifies the regulation ability of the *i*th host complex on the *j*th host gene; $-\lambda_{j}^{H}$ and $\delta_{j}^{H}$ indicate the degradation rate and the basal level of the *j*th host gene, respectively. In general, the basal level $\delta_{j}^{H}$ in Eq. 2 denotes an unknown regulation other than those mentioned above such as DNA methylation. $\varepsilon_{j}^{H}\left(t\right)$ represents the stochastic noise due to modeling residue at time *t*. Notably, in the case of the regulation ability $x_{j\left(I_{j}^{\prime \prime }\left(i^{\prime }-1\right)+i^{\prime \prime }\right)}^{H}$ of the *i*th host complex on the *j*th host gene, the index $I_{j}^{\prime \prime }\left(i^{\prime }-1\right)+i^{\prime \prime }$ guarantees the proper coordinate of the regulation ability $x_{j\left(I_{j}^{\prime \prime }\left(i^{\prime }-1\right)+i^{\prime \prime }\right)}^{H}$ of the *i*th host complex $p_{i^{\prime }}^{H}\left(t\right)p_{i^{\prime \prime }}^{H}\left(t\right)$ in the host GRN system matrix of the *j*th host gene, i.e., the regulation abilities of host complexes on the *j*th host gene can be aligned to an one row matrix of $x_{j1}^{H},\ldots ,x_{j\left(I_{j}^{\prime \prime }\right)}^{H},x_{j\left(I_{j}^{\prime \prime }+1\right)}^{H},\ldots ,x_{j\left(2I_{j}^{\prime \prime }\right)}^{H},x_{j\left(2I_{j}^{\prime \prime }+1\right)}^{H},\ldots ,x_{j\left(I_{j}^{\prime \prime }\left(i^{\prime }-1\right)+i^{\prime \prime }\right)}^{H},\ldots ,x_{j\left(I_{j}^{\prime \prime }I_{j}^{\prime }\right)}^{H}$.

Therefore, the biological meaning of Eq. 2 is that the expression level of the *j*th host gene can be regulated by numerous molecular mechanisms, including the host TF regulations $\sum_{i=1}^{I_{j}}b_{\mathrm{ji}}^{H}p_{i}^{H}\left(t\right)$ host lncRNA regulations $\sum_{n=1}^{N_{j}}e_{\mathrm{jn}}^{H}l_{n}^{H}\left(t\right)$, host complex regulations $\overset{I_{j}^{\prime }}{\underset{i^{\prime }=1}{\sum }}\overset{I_{j}^{\prime \prime }}{\underset{i^{\prime \prime }=1}{\sum }}x_{j\left(I_{j}^{\prime \prime }\left(i^{\prime }-1\right)+i^{\prime \prime }\right)}^{H}p_{i^{\prime }}^{H}\left(t\right)p_{i^{\prime \prime }}^{H}\left(t\right)$, host miRNA repressions $-\;\sum_{k=1}^{K_{j}}d_{\mathrm{jk}}^{H}g_{j}^{H}\left(t\right)m_{k}^{H}\left(t\right)$, mRNA degradation effect $-\lambda_{j}^{H}g_{j}^{H}\left(t\right)$, basal level $\delta_{j}^{H}$, and the corruption of stochastic noise $\varepsilon_{j}^{H}\left(t\right)$. Similar to protein model, the miRNA repression ability and gene degradation rate should be constrained to be non-positive. Since DNA methylation can directly influence the binding affinities of RNA polymerase to target genes (47), we assumed that the regulation by methyltransferase could cause the significant change of the basal level $\delta_{j}^{H}$ and the change of $\delta_{j}^{H}$ between the early stage and late stage of CDI in the dynamic model (2) implies the occurrence of methylation at the *j*th host gene in the infection process.

In Eq. 2, the expression of the *k*th host miRNA $m_{k}^{H}\left(t\right)$ and the *n*th host lncRNA $l_{n}^{H}\left(t\right)$ at time *t* can also be regulated by other regulators. Therefore, the dynamic regulatory equation of the *k*th host miRNA was modeled as follows:

(3) $$\begin{array}{l} m_{k}^{H}\left(t+1\right)=m_{k}^{H}\left(t\right)+\overset{I_{k}}{\underset{i=1}{\sum }}y_{\mathrm{ki}}^{H}p_{i}^{H}\left(t\right)-\mu_{k}^{H}m_{k}^{H}\left(t\right)+\varphi_{k}^{H}+\varsigma_{k}^{H}\left(t\right),\\ \;\text{for }k=1,2,\ldots ,K\text{ and }-\mu_{k}^{H}\leq 0 \end{array}$$

where $m_{k}^{H}\left(t\right)$ and $p_{i}^{H}\left(t\right)$ denote the expression levels of the *k*th host miRNA and the *i*th host TF at time *t*, respectively; $y_{\mathrm{ki}}^{H}$ represents the regulatory ability of the *i*th host TF on the *k*th host miRNA; *I_k_* signifies the number of host TFs that regulate the expression level of the *k*th host miRNA; $-\mu_{k}^{H}$ and $\varphi_{k}^{H}$ indicate the miRNA degradation rate and the basal level of the *k*th host miRNA, respectively; and $\varsigma_{k}^{H}\left(t\right)$ implies the stochastic noise at time *t*. The dynamic model of host miRNAs in Eq. 3 characterizes molecular regulatory mechanisms, including the transcription regulations $\sum_{i=1}^{\mathrm{Ik}}y_{\mathrm{ki}}^{H}p_{i}^{H}\left(t\right)$, miRNA degradation effect $-\mu_{k}^{H}m_{k}^{H}\left(t\right)$, basal level $\varphi_{k}^{H}$, and the corruption of stochastic noise $\varsigma_{k}^{H}\left(t\right)$. In addition, the degradation rate should be constrained to be non-positive.

Similarly, the dynamic model of the *n*th host lncRNA in candidate GEIN can be described by the dynamic equation as follows:

(4) $$\begin{array}{l} l_{n}^{H}\left(t+1\right)=l_{n}^{H}\left(t\right)+\overset{I_{n}}{\underset{i=1}{\sum }}z_{\mathrm{ni}}^{H}p_{i}^{H}\left(t\right)-\chi_{n}^{H}l_{n}^{H}\left(t\right)+\rho_{n}^{H}+\vartheta_{n}^{H}\left(t\right),\\ \;\text{for }n=1,2,\ldots ,N\text{ and}-\chi_{n}^{H}\leq 0 \end{array}$$

where $l_{n}^{H}\left(t\right)$ and $p_{i}^{H}\left(t\right)$ represent the expression levels of the *n*th host lncRNA and the *i*th host TF at time *t*, respectively; $z_{\mathrm{ni}}^{H}$ denotes the regulation ability of the *i*th host TF on the *n*th host lncRNA; I_n_ signifies the number of host TFs regulating the expression level of the *n*th host lncRNA; $-\chi_{n}^{H}$ and $\rho_{n}^{H}$ indicate the degradation rate and the basal level of the *n*th host lncRNA, respectively; and $\vartheta_{n}^{H}\left(t\right)$ is the stochastic noise due to the modeling residue. The dynamic model of host lncRNAs in Eq. 4 characterizes molecular regulatory mechanisms, including the transcription regulations $\sum_{i=1}^{\mathrm{In}}z_{\mathrm{ni}}^{H}p_{i}^{H}\left(t\right)$, degradation effect $-\chi_{n}^{H}l_{n}^{H}\left(t\right)$, basal level $\rho_{n}^{H}$, and the corruption of stochastic noise $\vartheta_{n}^{H}\left(t\right)$. Furthermore, the constraint of this model is that the lncRNA degradation rate should be non-positive.

For the PPIN of pathogen proteins in candidate GEIN, the dynamic model of the *q*th pathogen protein can be described by the following equation:

(5) $$\begin{array}{l} p_{q}^{P}\left(t+1\right)=p_{q}^{P}\left(t\right)+\overset{O_{q}}{\underset{o=1}{\sum }}a_{\mathrm{qo}}^{P}p_{q}^{P}\left(t\right)p_{o}^{P}\left(t\right)+\overset{I_{q}}{\underset{i=1}{\sum }}c_{\mathrm{qi}}^{P}p_{q}^{P}\left(t\right)p_{i}^{H}\left(t\right)+\alpha_{q}^{P}g_{q}^{P}\left(t\right)\\ \;-\gamma_{q}^{P}p_{q}^{P}\left(t\right)+\kappa_{q}^{P}+\varpi_{q}^{P}\left(t\right),\text{for }q=1,2,\ldots ,Q,\alpha_{q}^{P}\geq 0\text{ and }-\;\gamma_{q}^{P}\leq 0 \end{array}$$

where $p_{q}^{P}\left(t\right)$, $p_{o}^{P}\left(t\right)$, $g_{q}^{P}\left(t\right)$, and $p_{i}^{H}\left(t\right)$ represent the expression level of the *q*th pathogen protein, the *o*th pathogen protein, the *q*th pathogen gene, and the *i*th host protein at time *t*, respectively; $a_{\mathrm{qo}}^{P}$ and $c_{\mathrm{qi}}^{P}$ denote the interactive ability between the *q*th pathogen protein and *o*th pathogen protein, and between the *q*th pathogen protein and *i*th host protein, respectively; *O*_*q*_ and *I*_*q*_ signify the number of pathogen proteins and host proteins that interact with the *q*th pathogen protein, respectively; $\alpha_{q}^{P}$, $-\gamma_{q}^{P}$ and $\kappa_{q}^{P}$ indicate the translation rate, the degradation effect and the basal level of the *q*th pathogen protein, respectively; and $\varpi_{q}^{P}\left(t\right)$ denotes the stochastic noise of the *q*th pathogen protein at time *t*. The biological meaning of the Eq. 5 is that the expression level of the *q*th pathogen protein can be affected by various molecular interactive mechanisms, including the intraspecies PPIs $\sum_{o=1}^{\mathrm{Oq}}a_{\mathrm{qo}}^{P}p_{q}^{P}\left(t\right)p_{o}^{P}\left(t\right)$, the interspecies PPIs $\sum_{i=1}^{\mathrm{Iq}}c_{\mathrm{qi}}^{P}p_{q}^{P}\left(t\right)p_{i}^{H}\left(t\right)$, protein translation $\alpha_{q}^{P}g_{q}^{P}\left(t\right)$, protein degradation $-\gamma_{q}^{P}p_{q}^{P}\left(t\right)$, basal level $\kappa_{q}^{P}$, and the corruption of stochastic noise $\varpi_{q}^{P}\left(t\right)$. Similar to the host protein dynamic model, the translation rate should be constrained to be non-negative and the protein degradation rate should be constrained to be non-positive.

For the GRN of pathogen genes in candidate GEIN, the dynamic model of the *h*th pathogen gene can be described as follows:

(6) $$\begin{array}{l} g_{h}^{P}\left(t+1\right)=g_{h}^{P}\left(t\right)+\overset{Q_{h}}{\underset{q=1}{\sum }}b_{\mathrm{hq}}^{P}p_{q}^{P}\left(t\right)-\overset{K_{h}}{\underset{k=1}{\sum }}d_{\mathrm{hk}}^{P}g_{h}^{P}\left(t\right)m_{k}^{H}\left(t\right)\\ \;-\lambda_{h}^{P}g_{h}^{P}\left(t\right)+\delta_{h}^{P}+\varepsilon_{h}^{P}\left(t\right),\text{for }h=1,2,\ldots ,H,\;-d_{\mathrm{hk}}^{P}\leq 0\text{ and }-\lambda_{h}^{P}\leq 0 \end{array}$$

where $g_{h}^{P}\left(t\right)$, $p_{q}^{P}\left(t\right)$, and $m_{k}^{H}\left(t\right)$ indicate the expression levels of the *h*th pathogen gene, the *q*th pathogen TF, and the *k*th host miRNA at time *t*, respectively; $b_{\mathrm{hq}}^{P}$ and $-d_{\mathrm{hk}}^{P}$ represent the regulatory ability of the *q*th pathogen TF and the *k*th host miRNA on the *h*th pathogen gene, respectively; *Q*_*h*_ and *K*_*h*_ denote the number of pathogen TFs and host miRNAs that regulate the *q*th pathogen gene; $-\lambda_{h}^{P}$ and $\delta_{h}^{P}$ indicate the degradation rate and the basal level of the *q*th pathogen gene, respectively; and $\varepsilon_{h}^{P}\left(t\right)$ represent the stochastic noise due to the modeling residue at time *t*. The biological meaning of Eq. 6 is that the expression level of the *h*th pathogen gene can be regulated by various molecular mechanisms, including the pathogen TF regulations $\sum_{q=1}^{\mathrm{Qh}}b_{\mathrm{hq}}^{P}p_{q}^{P}\left(t\right)$, host miRNA repressions $-\;\sum_{k=1}^{\mathrm{Kh}}d_{\mathrm{hk}}^{P}g_{h}^{P}\left(t\right)m_{k}^{H}\left(t\right)$, mRNA degradation $-\lambda_{h}^{P}g_{h}^{P}\left(t\right)$, basal level $\delta_{h}^{P}$, and the corruption of stochastic noise $\varepsilon_{h}^{P}\left(t\right)$. In addition, the host-miRNA repression rate and degradation rate should be constrained to be non-positive. By comparing to the well-proposed models in the cross-talk genome-wide GEINs (48), this study considered additional molecular mechanisms in the GEIN, including transcription regulations of host lncRNA and host protein complex in Eq. 2 and the dynamic models of host miRNA in Eq. 3 and host lncRNA in Eq. 4, to characterize molecular interaction during cell infection in more detail.

### Parameter Estimation of the Dynamic Models of Candidate GEIN *via* the System Identification Method

In order to identify the precise parameters, we applied a system identification method to the dynamic genetic-and-epigenetic Eqs 1–6 in the candidate GEIN. We rewrote the host PPIN dynamic Eq. 1 as the linear regression form below,

(7) $$\begin{array}{l} p_{i}^{H}\left(t+1\right)=\left[\begin{array}{cccc} p_{i}^{H}\left(t\right)p_{1}^{H}\left(t\right) & \cdots & p_{i}^{H}\left(t\right)p_{F_{i}}^{H}\left(t\right) & p_{i}^{H}\left(t\right)p_{1}^{P}\left(t\right) \end{array}\right.\\ \begin{array}{cccc} \cdots & p_{i}^{H}\left(t\right)p_{Q_{i}}^{P}\left(t\right) & g_{i}^{H}\left(t\right) & \begin{array}{cc} p_{i}^{H}\left(t\right) & \left.1\right] \end{array} \end{array}\left[\begin{array}{c} a_{i1}^{H}\\ \vdots \\ a_{iF_{i}}^{H}\\ c_{i1}^{H}\\ \vdots \\ c_{iQ_{i}}^{H}\\ \alpha_{i}^{H}\\ 1-\gamma_{i}^{H}\\ \kappa_{i}^{H} \end{array}\right]+\varpi_{i}^{H}\left(t\right)\\ ≜\phi_{i}^{\mathrm{HP}}\left(t\right)\theta_{i}^{\mathrm{HP}}+\varpi_{i}^{H}\left(t\right),\text{for }i=1,2,\ldots ,I \end{array}$$

where $\phi_{i}^{\mathrm{HP}}\left(t\right)$ represents the regression vector that can be obtained from the microarray expression data and $\theta_{i}^{\mathrm{HP}}$ is the unknown parameter vector to be estimated for the *i*th host protein in host PPIN.

Equation 7 of the *i*th host protein can be augmented for *T_i_* data points as the following form:

(8) $$\left[\begin{array}{c} p_{i}^{H}\left(t_{2}\right)\\ p_{i}^{H}\left(t_{3}\right)\\ \vdots \\ \;p_{i}^{H}\left(t_{T_{i}}+1\right) \end{array}\right]\;=\;\left[\begin{array}{c} \phi_{i}^{\mathrm{HP}}\left(t_{1}\right)\\ \phi_{i}^{\mathrm{HP}}\left(t_{2}\right)\\ \vdots \\ \phi_{i}^{\mathrm{HP}}\left(t_{T_{i}}\right) \end{array}\right]\;\theta_{i}^{\mathrm{HP}}+\;\left[\begin{array}{c} \varpi_{i}^{H}\left(t_{1}\right)\\ \varpi_{i}^{H}\left(t_{2}\right)\\ \vdots \\ \varpi_{i}^{H}\left(t_{T_{i}}\right) \end{array}\right]\;\;,\text{ for }i=1,2,\ldots ,I$$

which could be simply represented as follows:

(9) $$P_{i}^{H}=\Phi_{i}^{\mathrm{HP}}\theta_{i}^{\mathrm{HP}}+\Gamma_{i}^{\mathrm{HP}},\text{ for }i=1,2,\ldots I$$

where $P_{i}^{H}=\;\left[\begin{array}{c} p_{i}^{H}\left(t_{2}\right)\\ p_{i}^{H}\left(t_{3}\right)\\ \vdots \\ \;p_{i}^{H}\left(t_{T_{i}}+1\right) \end{array}\right]\;,\;\Phi_{i}^{\mathrm{HP}}=\;\left[\begin{array}{c} \phi_{i}^{\mathrm{HP}}\left(t_{1}\right)\\ \phi_{i}^{\mathrm{HP}}\left(t_{2}\right)\\ \vdots \\ \phi_{i}^{\mathrm{HP}}\left(t_{T_{i}}\right) \end{array}\right]\;,\;\Gamma_{i}^{\mathrm{HP}}=\;\left[\begin{array}{c} \varpi_{i}^{H}\left(t_{1}\right)\\ \varpi_{i}^{H}\left(t_{2}\right)\\ \vdots \\ \varpi_{i}^{H}\left(t_{T_{i}}\right) \end{array}\right]$.

Therefore, the parameters in the vector $\theta_{i}^{\mathrm{HP}}$ can be estimated by applying the following constrained least-squares estimation problem,

(10) $$\begin{array}{ll} & \underset{\theta_{i}^{\mathrm{HP}}}{\min}\;{∥\Phi_{i}^{\mathrm{HP}}\theta_{i}^{\mathrm{HP}}-P_{i}^{H}∥}_{2}^{2}\\ & \text{subject to }\left[\begin{array}{ccccccccc} 0 & \cdots & 0 & 0 & \cdots & 0 & -1 & 0 & 0\\ 0 & \cdots & 0 & 0 & \cdots & 0 & 0 & 1 & 0 \end{array}\right]{\;\theta }_{i}^{\mathrm{HP}}\leq \left[\begin{array}{c} 0\\ 1 \end{array}\right] \end{array}$$

The parameters in the host PPIN dynamic Eq. 1 can be estimated by solving the constrained least-squares problem (10) *via* the help of *lsqlin* function in MATLAB optimization toolbox, and simultaneously the host protein translation rate $\alpha_{i}^{H}$ is guaranteed to be non-negative and the host protein degradation $-\gamma_{i}^{H}$ is guaranteed to be non-positive, i.e., $\alpha_{i}^{H}\geq 0$ and $-\gamma_{i}^{H}\leq 0$. Similarly, the constrained least-squares estimation problems of Eqs 2–6 are shown in supplementary methods.

As mentioned above, to avoid the overfitting problem in the parameter identification process, we have applied cubic spline to interpolate extra data points (five times number of the parameters in the parameter vector to be estimated, i.e., $\theta_{I}^{\mathrm{HP}}$ in host PPIN, $\theta_{j}^{\mathrm{HG}}$ in host GRN, $\theta_{k}^{\mathrm{HM}}$ in host miRNA dynamic model, $\theta_{n}^{\mathrm{HL}}$ in host lncRNA dynamic model, $\theta_{q}^{\mathrm{PP}}$ in pathogen PPIN, and $\theta_{h}^{\mathrm{PG}}$ in pathogen GRN). Therefore, with the microarray expression data, we could solve the constrained least-squares estimation problems in Eq. 10 and Eqs S3, S6, S9, S12, and S15 in Supplementary Material and identify the precise parameters in GEINs gene by gene (or protein by protein) *via* the *lsqlin* function of MATLAB optimization toolbox. Since the measurement technology of genome-wide protein expression of Caco-2 cells and *C. difficile* has not yet been realized, and about 73% variance of protein abundance can be explained by the corresponding mRNA abundance (49), the microarray data of gene expressions can replace protein expressions, providing sufficient information for solving above constrained least-squares parameter estimation problems.

## Author Contributions

C-WL and M-HS are co-first author. C-WL and M-HS: data analysis and interpretation, manuscript writing, methodology development, conception and design, data analysis and interpretation, manuscript writing. C-WL and B-SC: methodology development, conception and design, data analysis and interpretation, manuscript writing. C-WL and B-SC reviewed the paper, prepared figures, wrote and improved the scientific quality of the manuscript. All authors read and approved the final manuscript.

## Conflict of Interest Statement

The authors declare that the research was conducted in the absence of any commercial or financial relationships that could be construed as a potential conflict of interest.
