# Supplementary material for: Investigation of the Cross-talk Mechanism in Caco-2 Cells during Clostridium difficile Infection through Genetic-and-Epigenetic Interspecies Networks: Big Data Mining and Genome-Wide Identification
Source: Front Immunol. 2017 Aug 2;8:901. doi: 10.3389/fimmu.2017.00901 (PMC5539260; doi:10.3389/fimmu.2017.00901)
Supplement: Supplementary file 1 [file Data_Sheet_1.PDF]

Supplementary materials

Supplementary methods

Big data mining and data preprocessing of host/pathogen gene/miRNA microarray data

To identify the cross-talk activities between the host and pathogen during infection, it is necessary to simultaneously measure the gene expression of the host and pathogen. However, *C. difficile* is notoriously difficult to cultivate and isolate, and is extremely sensitive to oxygen, let alone culture with human cells in an anaerobic environment. There are only few studies investigating the gene expression of epithelial cells exposed to *C. difficile* toxins (1) and there is no existing transcription profile of *C. difficile* during the invasion to human cells except the raw data of one study (2). Therefore, in the present study, the raw data obtained from the previous study investigating the transcription profile of both Caco-2 cells and *C. difficile* (2) is the only available dataset providing sufficient two-sided information for constructing the candidate GEIN.

The microarray raw data has two parts. The first one contains the mRNA/miRNA expression profiles of three biological replicates of the Caco-2 cell line at 0, 30, 60, 120 min post-infection with *C. difficile* 630. Each biological replicate contains two technical replicates (GEO accession number GSE18407). The Caco-2 cell line was cultured in Dulbecco's modified Eagle's medium (DMEM) at 37°C prior to infection. The second part contains the mRNA expression profiles of three biological replicates of *C. difficile* 630 in Caco-2 cells at 0, 30, 60, 120 min post-infection (GEO accession number GSE18407; https://www.ncbi.nlm.nih.gov/geo/query/acc.cgi?acc=GSE18407). Furthermore, biological replicates 1, 2, and 3 of Caco-2 cells correspond to the biological replicates 1, 2, and 3 of *C. difficile*, respectively. The platforms used in the host and pathogen were Phalanx Human OneArray and *Clostridium difficile* 630/QCD32g58 array, respectively, which

include 39,200 and 13,824 probes, respectively. The microarray data were validated using qRT-PCR. Considering the fact that each biological replicate of Caco-2 cell contains two technical replicates, we then took the average of the microarray data of the two technical replicates for further network identification. The expression data of human Caco-2 cells at 0, 30, 60 min and *C. difficile* at 0, 30, 60 min during infection were utilized to identify the real GEINs of each of biological replicates during the early stage (0–60 min) of CDI. In addition, the expression data of human Caco-2 cells at 30, 60, 120 min and *C. difficile* at 30, 60, 120 min during infection were utilized to identify the real GEINs of each of biological replicates during the late stage (30–120 min) of CDI, where biological replicates 1, 2, and 3 in the early stage of infection correspond to biological replicate 1, 2, and 3 in the late stage of infection, respectively. To obtain a sufficient number of data points for the following network identification method, we applied the cubic spline interpolation method to the expression data at 0, 30, 60, 120 min to avoid an overfitting problem in the network identification process.

Construction of a candidate GEIN

In order to construct the candidate GEIN, big data collected from experimental or computational predictions are required. The source databases and literature of the big data are as follows. The required information on host candidate PPIN protein-protein interaction (PPI) was obtained from DIP (3), BIND (4), IntAct (5), MINT (6) and the physical interaction part of BioGRID (7) since the genetic interactions of BioGRID are inferred indirectly from experiments. The host candidate GRN required of the TFs/lncRNAs/complex and their downstream-regulated genes was obtained from HTRIdb (8) and ITFP (9). miRNAs to regulatory gene associations were from TargetScanHuman

(10), starBase v2 (11) and CircuitDB (12). The pathogen candidate PPIN required of PPI information can be obtained from STRING (13). In the case of pathogen candidate GRN and host-pathogen cross-talk candidate PPIN and GRN, no existing database can provide sufficient information or predictions for network construction. Thus, we explored studies reporting host-pathogen interspecies PPIs (14-27), and utilized the sequence homology between *C. difficile* and *Escherichia coli* (*E. coli*) (28), as well as *C. difficile* with *Homo sapiens* (28), along with reported interspecies PPIN between *E. coli* and *Homo sapiens* (29), and human intraspecies PPIs (DIP, BIND, BioGRID, IntAct and MINT), to construct the host-pathogen candidate interspecies PPIN. Similarly, we surveyed the literature reporting pathogen gene regulatory pairs (30-39), and utilized the sequence homology between *C. difficile* and *E. coli* (28), as well as *C. difficile* and *Homo sapiens* (28), along with the *E. coli* intraspecies GRN obtained from RegulonDB (40) and human intraspecies GRN (HTRIdb and ITFP), to build the pathogen candidate intraspecies GRN. Finally, we utilized the sequence homology between *C. difficile* and *Homo sapiens*, along with the miRNA regulatory information obtained from TargetScanHuman (10), starBase v2 (11) and CircuitDB (12) to create the candidate GRN of host-miRNAs targeting pathogen-genes. The detailed construction procedure of host-pathogen candidate interspecies PPIN, pathogen candidate intraspecies GRN, and candidate GRN of host-miRNAs targeting pathogen-genes, are shown in S6 Fig.

We obtained 144,361 TF/lncRNA/complex gene pairs, 5,961 miRNA gene pairs for host candidate GRN, 1,265 TF gene pairs and 96 miRNA gene pairs for pathogen candidate GRN. In the case of PPINs, we inferred 3,425,976 PPIs for the host candidate PPIN, 290,018 PPIs for pathogen candidate PPIN, and 17,068 interspecies candidate PPIs

between host and pathogen during *C. difficile* infection.

Parameter estimation of the dynamic models of candidate GEIN via the system identification method

Similarly, we rewrote the host GRN dynamic equation (2) as the linear regression form below,

$$
\begin{aligned}
g_j^H(t+1) = \Big[& p_1^H(t) \ \cdots \ p_{I_j}^H(t) \ \ l_1^H(t) \ \cdots \ l_{N_j}^H(t) \\
& p_1^H(t)p_1^H(t) \ \cdots \ p_{I'_j}^H(t)p_{I''_j}^H(t) \ \ g_j^H(t)m_1^H(t) \ \cdots \\
& g_j^H(t)m_{K_j}^H(t) \ \ g_j^H(t) \ \ 1 \Big]
\begin{bmatrix} b_{j1}^H \\ \vdots \\ b_{jI_j}^H \\ e_{j1}^H \\ \vdots \\ e_{jN_j}^H \\ x_{j1}^H \\ \vdots \\ x_{j(I''_j \times I''_j)}^H \\ -d_{j1}^H \\ \vdots \\ -d_{jK_j}^H \\ 1-\lambda_j^H \\ \delta_j^H \end{bmatrix} + \varepsilon_j^H(t) \\
\triangleq \ & \phi_j^{HG}(t)\theta_j^{HG} + \varepsilon_j^H(t), \ \text{for}\ j = 1,2,\ldots,J
\end{aligned}
\qquad \text{(S1)}
$$

where ϕ_j^{HG}(t) represents the regression vector that can be obtained from the microarray expression data, and θ_j^{HG} is the unknown parameter vector to be estimated for the *j*th host gene in host GRN.

Equation (S1) of the *j*th host gene can be augmented for T_j data points as the following form,

$$
G_j^H = \Phi_j^{HG}\theta_j^{HG} + \Gamma_j^{HG}, \ \text{for}\ j = 1,2,\ldots,J \qquad \text{(S2)}
$$

where $G_j^H = \begin{bmatrix} g_j^H(t_2) \\ g_j^H(t_3) \\ \vdots \\ g_j^H(t_{T_j}+1) \end{bmatrix}$, $\Phi_j^{HG} = \begin{bmatrix} \phi_j^{HG}(t_1) \\ \phi_j^{HG}(t_2) \\ \vdots \\ \phi_j^{HG}(t_{T_j}) \end{bmatrix}$, $\Gamma_j^{HG} = \begin{bmatrix} \varepsilon_j^H(t_1) \\ \varepsilon_j^H(t_2) \\ \vdots \\ \varepsilon_j^H(t_{T_j}) \end{bmatrix}$.

Therefore, the parameters in the vector θ_j^{HG} can be estimated by applying the following constrained least-squares estimation problem,

$$\begin{aligned} &\min_{\theta_j^{HG}} \left\| \Phi_j^{HG}\theta_j^{HG} - G_j^H \right\|_2^2 \\ &\text{subject to} \begin{bmatrix} 0 & \cdots & 0 & 0 & \cdots & 0 & 0 & \cdots & 0 & 1 & 0 & \cdots & \cdots & 0 \\ \vdots & \ddots & \vdots & \vdots & \ddots & \vdots & \vdots & \ddots & \vdots & 0 & \ddots & \ddots & \cdots & \vdots \\ 0 & \cdots & 0 & 0 & \cdots & 0 & 0 & \cdots & 0 & \vdots & \ddots & 1 & \ddots & \vdots \\ 0 & \cdots & 0 & 0 & \cdots & 0 & 0 & \cdots & 0 & 0 & \cdots & 0 & 1 & 0 \end{bmatrix} \theta_j^{HG} \le \begin{bmatrix} 0 \\ \vdots \\ 0 \\ 1 \end{bmatrix} \end{aligned} \tag{S3}$$

The parameters in the host GRN dynamic equation (2) can be estimated by solving the above constrained optimization problem (S3), and simultaneously the host miRNA repression -d_{jk}^H is guaranteed to be non-positive and the host gene degradation -λ_j^H is guaranteed to be non-positive, i.e. -$d_{jk}^H \le 0$ for $k = 1, \ldots, K_j$ and -$\lambda_j^H \le 0$.

The dynamic model of host miRNAs in equation (3) can also be rewritten as the linear regression form below,

$$\begin{aligned} m_k^H(t+1) &= \begin{bmatrix} p_1^H(t) & \cdots & p_{I_k}^H(t) & m_k^H(t) & 1 \end{bmatrix} \begin{bmatrix} y_{k1}^H \\ \vdots \\ y_{kI_k}^H \\ 1-\mu_k^H \\ \varphi_k^H \end{bmatrix} + \varsigma_k^H(t) \\ &\triangleq \phi_k^{HM}(t)\theta_k^{HM} + \varsigma_k^H(t), \text{ for } k = 1,2,\ldots,K \end{aligned} \tag{S4}$$

where ϕ_k^{HM}(t) represents the regression vector that can be obtained from the microarray expression data, and θ_k^{HM} is the unknown parameter vector to be estimated for the kth host miRNA in host GRN.

Equation (S4) of the kth host miRNA can be augmented for T_k data points as the following

form,

$$M_k^H = \Phi_k^{HM}\theta_k^{HM} + \Gamma_k^{HM}, \text{ for } k = 1,2,\ldots,K \tag{S5}$$

where $M_k^H = \begin{bmatrix} m_k^H(t_2) \\ m_k^H(t_3) \\ \vdots \\ m_k^H(t_{T_k}+1) \end{bmatrix}$, $\Phi_k^{HM} = \begin{bmatrix} \phi_k^{HM}(t_1) \\ \phi_k^{HM}(t_2) \\ \vdots \\ \phi_k^{HM}(t_{T_k}) \end{bmatrix}$, $\Gamma_k^{HM} = \begin{bmatrix} \varsigma_k^H(t_1) \\ \varsigma_k^H(t_2) \\ \vdots \\ \varsigma_k^H(t_{T_k}) \end{bmatrix}$.

Therefore, the parameters in the vector θ_k^{HM} can be estimated by applying the following constrained least-squares estimation problem,

$$\begin{aligned} &\min_{\theta_k^{HM}} \left\| \Phi_k^{HM}\theta_k^{HM} - M_k^H \right\|_2^2 \\ &\text{subject to } \begin{bmatrix} 0 & \cdots & 0 & 1 & 0 \end{bmatrix} \theta_k^{HM} \le 1 \end{aligned} \tag{S6}$$

The parameters in the host miRNA dynamic equation (3) can be estimated by solving the constrained least-squares problem (S6), and simultaneously the host miRNA degradation rate $-\mu_k^H$ is guaranteed to be non-positive, i.e. $-\mu_k^H \le 0$.

Similarly, we rewrote the dynamic model of host lncRNAs in equation (4) as the following linear regression form,

$$\begin{aligned} l_n^H(t+1) &= \begin{bmatrix} p_1^H(t) & \cdots & p_{I_n}^H(t) & l_n^H(t) & 1 \end{bmatrix} \begin{bmatrix} z_{n1}^H \\ \vdots \\ z_{nI_n}^H \\ 1-\chi_n^H \\ \rho_n^H \end{bmatrix} + \vartheta_n^H(t) \\ &\triangleq \phi_n^{HL}(t)\theta_n^{HL} + \vartheta_n^H(t), \text{ for } n = 1,2,\ldots,N \end{aligned} \tag{S7}$$

where ϕ_n^{HL} (t) denotes the regression vector that can be obtained from the microarray expression data, and θ_n^{HL} is the unknown parameter vector to be estimated for the *n*th host lncRNA in host GRN.

Equation (S7) of the *n*th host lncRNA can be augmented for T_n data points as the following form,

$$L_n^H = \Phi_n^{HL}\theta_n^{HL} + \Gamma_n^{HL}, \text{ for } n = 1,2,\ldots,N \tag{S8}$$

where $L_n^H = \begin{bmatrix} l_n^H(t_2) \\ l_n^H(t_3) \\ \vdots \\ l_n^H(t_{T_n}+1) \end{bmatrix}$, $\Phi_n^{HL} = \begin{bmatrix} \phi_n^{HL}(t_1) \\ \phi_n^{HL}(t_2) \\ \vdots \\ \phi_n^{HL}(t_{T_n}) \end{bmatrix}$, $\Gamma_n^{HL} = \begin{bmatrix} \vartheta_n^H(t_1) \\ \vartheta_n^H(t_2) \\ \vdots \\ \vartheta_n^H(t_{T_n}) \end{bmatrix}$.

Therefore, the parameters in the vector θ_n^{HL} can be estimated by applying the following constrained least-squares estimation problem,

$$\begin{aligned} &\min_{\theta_n^{HL}} \left\| \Phi_n^{HL}\theta_n^{HL} - L_n^H \right\|_2^2 \\ &\text{subject to } \begin{bmatrix} 0 & \cdots & 0 & 1 & 0 \end{bmatrix} \theta_n^{HL} \le 1 \end{aligned} \tag{S9}$$

The parameters in the host lncRNA dynamic equation (4) can be estimated by solving the constrained least-squares problem (S9), and simultaneously the host lncRNA degradation rate -χ_n^H is guaranteed to be non-positive, i.e. -$\chi_n^H \le 0$.

By the same process as in the host PPIN dynamic equation (1), we rewrote the pathogen PPIN equation (5) as the following linear regression form,

$$\begin{aligned} p_q^P(t+1) = &\Big[p_q^P(t)p_1^P(t) \quad \cdots \quad p_q^P(t)p_{O_q}^P(t) \quad p_q^P(t)p_1^H(t) \\ &\cdots \quad p_q^P(t)p_{I_q}^H(t) \quad g_q^P(t) \quad p_q^P(t) \quad 1 \Big] \begin{bmatrix} a_{q1}^P \\ \vdots \\ a_{qO_q}^P \\ c_{q1}^P \\ \vdots \\ c_{qI_q}^P \\ \alpha_q^P \\ 1-\gamma_q^P \\ \kappa_q^P \end{bmatrix} + \varpi_q^P(t) \\ &\triangleq \phi_q^{PP}(t)\theta_q^{PP} + \varpi_q^P(t), \text{ for } q = 1,2,\ldots,Q \end{aligned} \tag{S10}$$

where ϕ_q^{PP}(t) represents the regression vector that can be obtained from the microarray expression data, and θ_q^{PP} is the unknown parameter vector to be estimated for the qth

pathogen protein in pathogen PPIN.

Equation (S10) of the qth pathogen protein can be augmented for T_q data points as the following form,

$$P_q^P = \Phi_q^{PP}\theta_q^{PP} + \Gamma_q^{PP}, \text{ for } q = 1,2,\ldots,Q \tag{S11}$$

where $P_q^P = \begin{bmatrix} p_q^P(t_2) \\ p_q^P(t_3) \\ \vdots \\ p_q^P\left(t_{T_q}+1\right) \end{bmatrix}$, $\Phi_q^{PP} = \begin{bmatrix} \phi_q^{PP}(t_1) \\ \phi_q^{PP}(t_2) \\ \vdots \\ \phi_q^{PP}\left(t_{T_q}\right) \end{bmatrix}$, $\Gamma_q^{PP} = \begin{bmatrix} \varpi_q^P(t_1) \\ \varpi_q^P(t_2) \\ \vdots \\ \varpi_q^P\left(t_{T_q}\right) \end{bmatrix}$.

Therefore, the parameters in the vector θ_q^{PP} can be estimated by applying the following constrained least-squares estimation problem,

$$\begin{aligned} &\min_{\theta_q^{PP}} \left\| \Phi_q^{PP}\theta_q^{PP} - P_q^P \right\|_2^2 \\ &\text{subject to } \begin{bmatrix} 0 & \cdots & 0 & 0 & \cdots & 0 & -1 & 0 & 0 \\ 0 & \cdots & 0 & 0 & \cdots & 0 & 0 & 1 & 0 \end{bmatrix} \theta_q^{PP} \leq \begin{bmatrix} 0 \\ 1 \end{bmatrix} \end{aligned} \tag{S12}$$

The parameters in the pathogen PPIN dynamic equation (5) can be estimated by solving the constrained least-squares problem (S12), which could guarantee the pathogen protein translation rate α_q^P to be non-negative and the pathogen protein degradation rate -γ_q^P to be non-positive simultaneously, i.e. $\alpha_q^P \geq 0$ and -$\gamma_q^P \leq 0$.

Finally, we rewrote the pathogen GRN equation (6) as the linear regression form below,

$$g_h^P(t+1) = \left[p_1^P(t) \quad \cdots \quad p_{Q_h}^P(t) \quad g_h^P(t) m_1^H(t) \right.$$

$$\left. \cdots \quad g_h^P(t) m_{K_h}^H(t) \quad g_h^P(t) \quad 1 \right] \begin{bmatrix} b_{h1}^P \\ \vdots \\ b_{hQ_h}^P \\ -d_{h1}^P \\ \vdots \\ -d_{hK_h}^P \\ 1-\lambda_h^P \\ \delta_h^P \end{bmatrix} + \varepsilon_h^P(t) \qquad \text{(S13)}$$

$$\triangleq \phi_h^{PG}(t)\theta_h^{PG} + \varepsilon_h^P(t), \text{ for } h = 1,2,\ldots,H$$

where ϕ_h^{PG}(t) represents the regression vector that can be obtained from the microarray expression data, and θ_h^{PG} is the unknown parameter vector to be estimated for the *h*th pathogen gene in pathogen GRN.

Equation (S13) of the *h*th pathogen gene can be augmented for T_h data points as the following form,

$$G_h^P = \Phi_h^{PG}\theta_h^{PG} + \Gamma_h^{PG}, \text{ for } h = 1,2,\ldots,H \qquad \text{(S14)}$$

where $G_h^P = \begin{bmatrix} g_h^P(t_2) \\ g_h^P(t_3) \\ \vdots \\ g_h^P(t_{T_h}+1) \end{bmatrix}$, $\Phi_h^{PG} = \begin{bmatrix} \phi_h^{PG}(t_1) \\ \phi_h^{PG}(t_2) \\ \vdots \\ \phi_h^{PG}(t_{T_h}) \end{bmatrix}$, $\Gamma_h^{PG} = \begin{bmatrix} \varepsilon_h^P(t_1) \\ \varepsilon_h^P(t_2) \\ \vdots \\ \varepsilon_h^P(t_{T_h}) \end{bmatrix}$.

Thus, the parameters in the vector θ_h^{PG} can be estimated by applying the following constrained least-squares estimation problem,

$$\min_{\theta_h^{PG}} \left\| \Phi_h^{PG}\theta_h^{PG} - G_h^P \right\|_2^2$$

$$\text{subject to } \begin{bmatrix} 0 & \cdots & 0 & 1 & 0 & \cdots & \cdots & 0 \\ \vdots & \ddots & \vdots & 0 & \ddots & \ddots & \cdots & \vdots \\ 0 & \cdots & 0 & \vdots & \ddots & 1 & \ddots & \vdots \\ 0 & \cdots & 0 & 0 & \cdots & 0 & 1 & 0 \end{bmatrix} \theta_h^{PG} \le \begin{bmatrix} 0 \\ \vdots \\ 0 \\ 1 \end{bmatrix} \qquad \text{(S15)}$$

The parameters in the pathogen GRN dynamic equation (6) can be obtained by solving the

above constrained least-squares estimation problem (S15), which could guarantee the host miRNA repression -d_{hk}^{P} to be non-positive and the pathogen gene degradation effect -λ_h^P to be non-positive simultaneously, i.e. -$d_{hk}^{P}\leq 0$ and -$\lambda_h^P\leq 0$.

Remark.1 Note that the dynamic model of host miRNAs in equation (3), and host lncRNAs in equation (4) can be regulated by regulators other than host TFs such as host miRNAs and host lncRNAs. While in the candidate GEIN, no regulatory relationships about miRNA-to-miRNA, miRNA-to-lncRNA, lncRNA-to-miRNA and lncRNA-to-lncRNA was found by big data mining. Therefore, no corresponding terms exist in equations (3) and (4).

Remark.2 The epigenetic regulation of candidate GEIN in (41) only considered the regulation of miRNAs. In this study, the epigenetic regulation of candidate GEIN in (1)–(6) considered miRNAs, lncRNAs, acetylation, ubiquitination and methylation. Furthermore, the transcriptional regulation of miRNA and lncRNA genes via TFs allow us to construct the dynamic models of host miRNAs and lncRNAs as shown in (3) and (4), which are absent in (41). Therefore, the proposed GEIN in this study contains more epigenetic information for cellular mechanisms in infection than that in (41).

Pruning false-positives in candidate GEIN for real GEIN via system order detection scheme

Because a candidate GEIN contains several false-positive information obtained from computational, experimental and homology-dependent predictions, we need to apply a system order detection scheme to the network models in (1)–(6) to prune these false-positives in the candidate GEIN. Therefore, we applied Akaike information criterion (AIC)

to delete the insignificant parameters that were out of the system order in models of the candidate GEIN (42) using the real microarray data of Caco-2 cells and *C. difficile* in the early and late stage of infection. Next, we obtained the real GEINs in the early and late stage of CDI.

For the host PPIN model in (9), AIC of the *i*th host protein in the PPIN can be defined as the function of system interaction order as follows (42),

$$AIC_i^{HP}\left(F_i,Q_i\right)=\log\left(\frac{1}{T_i}\left(P_i^H-\Phi_i^{HP}\hat{\theta}_i^{HP}\right)^T\left(P_i^H-\Phi_i^{HP}\hat{\theta}_i^{HP}\right)\right)+\frac{2\left(F_i+Q_i\right)}{T_i} \tag{S16}$$

where $\hat{\theta}_i^{HP}$ represents the estimated parameter vector of the *i*th host protein by solving the constrained optimization problem in (10); and $\hat{\sigma}_i^{HP,2}=\frac{1}{T_i}\left(P_i^H-\Phi_i^{HP}\hat{\theta}_i^{HP}\right)^T\left(P_i^H-\Phi_i^{HP}\hat{\theta}_i^{HP}\right)$ is the estimated residual error. When we low down the system interaction order ($F_i + Q_i$), the corresponding residual error will increase. Likewise, when we attempt to minimize the residual error, the system order increases. Therefore, we need to tradeoff the residual error and the system order to achieve the minimum value of AIC_i^{HP} for real system order. Accordingly, the real system order $F_i^* + Q_i^*$ for the real host PPIN of GEIN could minimize $AIC_i^{HP}(F_i, Q_i)$ (41-43). Based on the theory of AIC, host proteins whose interaction abilities are out of F_i^* as well as pathogen proteins whose interaction abilities are out of Q_i^* should be considered as false positives and pruned away from the candidate PPIs to obtain the real PPIs one protein by one protein in GEIN.

Similarly, for the host GRN model in (S2), AIC of gene regulations in the *j*th host gene can be defined as follows,

$$AIC_j^{HG}\left(I_j,N_j,I'I'',K_j\right)=\log\left(\frac{1}{T_j}\left(G_j^H-\Phi_j^{HG}\hat{\theta}_j^{HG}\right)^T\left(G_j^H-\Phi_j^{HG}\hat{\theta}_j^{HG}\right)\right)+\frac{2\left(I_j+N_j+I'I''+K_j\right)}{T_j} \tag{S17}$$

where $\hat{\theta}_j^{HG}$ signifies the estimated parameter vector of the *j*th host gene by solving the constrained least-squares problem in (S3); and $\hat{\sigma}_j^{HG,2}=\frac{1}{T_j}\left(G_j^H-\Phi_j^{HG}\hat{\theta}_j^{HG}\right)^T\left(G_j^H-\Phi_j^{HG}\hat{\theta}_j^{HG}\right)$ is the estimated residual error. We then achieved the minimum value of AIC_j^{HG} to get the corresponding real system order $I_j^* + N_j^* + (I'I'')^* + K_j^*$ to prune the false positive regulations of candidate GRN one gene by one gene for the real host GRN of GEIN.

In addition, for the host miRNA model in (S5) and the lncRNA model in (S8), AICs of the *k*th host miRNA and the *n*th host lncRNA can be defined as follows, respectively,

$$AIC_k^{HM}\left(I_k\right)=\log\left(\frac{1}{T_k}\left(M_k^H-\Phi_k^{HM}\hat{\theta}_k^{HM}\right)^T\left(M_k^H-\Phi_k^{HM}\hat{\theta}_k^{HM}\right)\right)+\frac{2\left(I_k\right)}{T_k} \tag{S18}$$

$$AIC_n^{HL}\left(I_n\right)=\log\left(\frac{1}{T_n}\left(L_n^H-\Phi_n^{HL}\hat{\theta}_n^{HL}\right)^T\left(L_n^H-\Phi_n^{HL}\hat{\theta}_n^{HL}\right)\right)+\frac{2\left(I_n\right)}{T_n} \tag{S19}$$

where $\hat{\theta}_k^{HM}$ and $\hat{\theta}_n^{HL}$ denote the estimated parameter vector of the *k*th host miRNA by solving the constrained optimization problem in (S6) and the *n*th host lncRNA by solving the constrained optimization problem in (S9), respectively; $\hat{\sigma}_k^{HM,2}=\frac{1}{T_k}\left(M_k^H-\Phi_k^{HM}\hat{\theta}_k^{HM}\right)^T\left(M_k^H-\Phi_k^{HM}\hat{\theta}_k^{HM}\right)$ and $\hat{\sigma}_n^{HL,2}=\frac{1}{T_n}\left(L_n^H-\Phi_n^{HL}\hat{\theta}_n^{HL}\right)^T\left(L_n^H-\Phi_n^{HL}\hat{\theta}_n^{HL}\right)$ represent the estimated residual error of the *k*th

host miRNA and the nth host lncRNA, respectively. Thherefore, by the tradeoff between system order and residual error in the corresponding AIC, we can achieve the minimum AIC_k^{HM} and AIC_n^{HL} to obtain their corresponding real system order I_k^* and I_n^* to prune the false positives off I_k^* and I_n^* for the real host miRNA regulations and the real host lncRNA regulations of GEIN, respectively.

Finally, we applied the same pruning process by the AIC method as in host GEIN to prune the false-positives from pathogen candidate GEIN. For the pathogen protein interaction model in (S11) and the pathogen gene regulatory model in (S14), AIC of PPIs in the qth pathogen protein, and gene regulations of the hth pathogen gene can be defined as follows, respectively,

$$AIC_q^{PP}\left(O_q,I_q\right)=\log\left(\frac{1}{T_q}\left(P_q^P-\Phi_q^{PP}\hat{\theta}_q^{PP}\right)^T\left(P_q^P-\Phi_q^{PP}\hat{\theta}_q^{PP}\right)\right)+\frac{2\left(O_q+I_q\right)}{T_q} \tag{S20}$$

$$AIC_h^{PG}\left(Q_h,K_h\right)=\log\left(\frac{1}{T_h}\left(G_h^P-\Phi_h^{PG}\hat{\theta}_h^{PG}\right)^T\left(G_h^P-\Phi_h^{PG}\hat{\theta}_h^{PG}\right)\right)+\frac{2\left(Q_h+K_h\right)}{T_h} \tag{S21}$$

where $\hat{\theta}_q^{PP}$ and $\hat{\theta}_h^{PG}$ denote the estimated parameter vector of the qth pathogen protein by solving the constrained optimization problem in (S12), and the hth pathogen gene by solving the constrained optimization problem in (S15), respectively. $\hat{\sigma}_q^{PP,2}=\frac{1}{T_q}\left(P_q^P-\Phi_q^{PP}\hat{\theta}_q^{PP}\right)^T\left(P_q^P-\Phi_q^{PP}\hat{\theta}_q^{PP}\right)$ and $\hat{\sigma}_h^{PG,2}=\frac{1}{T_h}\left(G_h^P-\Phi_h^{PG}\hat{\theta}_h^{PG}\right)^T\left(G_h^P-\Phi_h^{PG}\hat{\theta}_h^{PG}\right)$ are the estimated residual error of the qth pathogen protein and the hth pathogen gene, respectively. Thus, by the tradeoff between system order and residual error in AIC, we achieved the minimum AIC_q^{PP} and AIC_h^{PG} to obtain their corresponding real system order $O_q^*+I_q^*$ and $Q_h^*+K_h^*$ to prune false positives

for the real PPIN and GRN of real pathogen GEIN, respectively.

After identifying the system order and pruning the false-positives of candidate GEINs, we finally got the real GEINs of the early and late stage of CDI for each replicate. Since the real GEINs are still very complex, it is difficult to investigate the precise host-pathogen interaction mechanisms from these networks. We therefore performed PNP method to extract the core network structures of these GEINs to help investigate the cross-talk mechanisms at different stages of CDI.

Extracting core network structures from real GEINs via PNP method

To prepare for applying PNP method to extract the host-pathogen core networks from real GEINs, we need to construct a combined network matrix H that contains all estimated parameters in the real GEIN as follows,

$$H = \begin{bmatrix} H_{hp,hp} & H_{hp,pp} & 0 & 0 & 0 \\ H_{pp,hp} & H_{pp,pp} & 0 & 0 & 0 \\ H_{hg,hp} & 0 & H_{hg,hm} & H_{hg,hl} & H_{hg,hc} \\ 0 & H_{pg,pp} & H_{pg,hm} & 0 & 0 \\ H_{hm,hp} & 0 & 0 & 0 & 0 \\ H_{hl,hp} & 0 & 0 & 0 & 0 \end{bmatrix} \in \mathbb{R}^{(2I+2Q+K+N)\times\left(I+Q+K+N+I^{'}I^{''}\right)}$$

where $H_{hp,hp}=\begin{bmatrix} \hat{a}_{11}^{H} & \cdots & \hat{a}_{1I}^{H} \\ \vdots & \hat{a}_{if}^{H} & \vdots \\ \hat{a}_{I1}^{H} & \cdots & \hat{a}_{II}^{H} \end{bmatrix}$; $H_{hp,pp}=\begin{bmatrix} \hat{c}_{11}^{H} & \cdots & \hat{c}_{1Q}^{H} \\ \vdots & \hat{c}_{iq}^{H} & \vdots \\ \hat{c}_{I1}^{H} & \cdots & \hat{c}_{IQ}^{H} \end{bmatrix}$; $H_{pp,hp}=\begin{bmatrix} \hat{c}_{11}^{P} & \cdots & \hat{c}_{1I}^{P} \\ \vdots & \hat{c}_{qi}^{P} & \vdots \\ \hat{c}_{Q1}^{P} & \cdots & \hat{c}_{QI}^{P} \end{bmatrix}$;

$H_{pp,pp}=\begin{bmatrix} \hat{a}_{11}^{P} & \cdots & \hat{a}_{1Q}^{P} \\ \vdots & \hat{a}_{qo}^{P} & \vdots \\ \hat{a}_{Q1}^{P} & \cdots & \hat{a}_{QQ}^{P} \end{bmatrix}$; $H_{hg,hp}=\begin{bmatrix} \hat{b}_{11}^{H} & \cdots & \hat{b}_{1I}^{H} \\ \vdots & \hat{b}_{ji}^{H} & \vdots \\ \hat{b}_{I1}^{H} & \cdots & \hat{b}_{II}^{H} \end{bmatrix}$; $H_{hg,hm}=\begin{bmatrix} -\hat{d}_{11}^{H} & \cdots & -\hat{d}_{1K}^{H} \\ \vdots & -\hat{d}_{jk}^{H} & \vdots \\ -\hat{d}_{I1}^{H} & \cdots & -\hat{d}_{IK}^{H} \end{bmatrix}$;

$H_{hg,hl}=\begin{bmatrix} \hat{e}_{11}^{H} & \cdots & \hat{e}_{1N}^{H} \\ \vdots & \hat{e}_{jn}^{H} & \vdots \\ \hat{e}_{I1}^{H} & \cdots & \hat{e}_{IN}^{H} \end{bmatrix}$; $H_{hg,hc}=\begin{bmatrix} \hat{x}_{11}^{H} & \cdots & \hat{x}_{1I'I''}^{H} \\ \vdots & \hat{x}_{j\left(I_j''\left(i'-1\right)+i''\right)}^{H} & \vdots \\ \hat{x}_{I1}^{H} & \cdots & \hat{x}_{II'I''}^{H} \end{bmatrix}$; $H_{pg,pp}=\begin{bmatrix} \hat{b}_{11}^{P} & \cdots & \hat{b}_{1Q}^{P} \\ \vdots & \hat{b}_{hq}^{P} & \vdots \\ \hat{b}_{Q1}^{P} & \cdots & \hat{b}_{QQ}^{P} \end{bmatrix}$;

$H_{pg,hm}=\begin{bmatrix} -\hat{d}_{11}^{P} & \cdots & -\hat{d}_{1K}^{P} \\ \vdots & -\hat{d}_{hk}^{P} & \vdots \\ -\hat{d}_{Q1}^{P} & \cdots & -\hat{d}_{QK}^{P} \end{bmatrix}$; $H_{hm,hp}=\begin{bmatrix} \hat{y}_{11}^{H} & \cdots & \hat{y}_{1I}^{H} \\ \vdots & \hat{y}_{ki}^{H} & \vdots \\ \hat{y}_{K1}^{H} & \cdots & \hat{y}_{KI}^{H} \end{bmatrix}$ and $H_{hl,\mathrm{hp}}=\begin{bmatrix} \hat{z}_{11}^{H} & \cdots & \hat{z}_{1I}^{H} \\ \vdots & \hat{z}_{ni}^{H} & \vdots \\ \hat{z}_{N1}^{H} & \cdots & \hat{z}_{NI}^{H} \end{bmatrix}$.

$\hat{a}_{if}^{H}$ and $\hat{c}_{iq}^{H}$ could be obtained from $\hat{\theta}_{i}^{HP}$ by solving the parameter estimation problem in (10) and pruning false positives by AIC method in (S16); $\hat{b}_{ji}^{H}$, $-\hat{d}_{jk}^{H}$, $\hat{e}_{jn}^{H}$ and $\hat{x}_{j\left(I_j''\left(i'-1\right)+i''\right)}^{H}$ could be obtained from $\hat{\theta}_{j}^{HG}$ by solving the parameter estimation problem in (S3) and pruning false positives by AIC method in (S17); $\hat{y}_{ki}^{H}$ could be obtained from $\hat{\theta}_{k}^{HM}$ by solving the parameter estimation problem in (S6) and pruning false positives by AIC method in (S18); $\hat{z}_{ni}^{H}$ could be obtained from $\hat{\theta}_{n}^{HL}$ by solving the parameter estimation problem in (S9) and pruning false positives by AIC method in (S19); $\hat{c}_{qi}^{P}$ and $\hat{a}_{qo}^{P}$ could be obtained from $\hat{\theta}_{q}^{PP}$ by solving the parameter estimation problem in (S12) and pruning false positives by AIC method in (S20); and $\hat{b}_{hq}^{P}$ and $-\hat{d}_{hk}^{P}$ could be obtained from $\hat{\theta}_{h}^{PG}$ by solving the parameter estimation problem in (S15) and pruning false positives by AIC method in (S21). $\hat{a}_{if}^{H}$ and $\hat{a}_{qo}^{P}$ represent the interactive abilities of intraspecies PPIs in host

and pathogen PPINs; $\hat{c}_{iq}^{H}$ and $\hat{c}_{qi}^{P}$ signify the interactive abilities between host protein and pathogen protein in the interspecies PPIN; $\hat{b}_{ji}^{H}$ and $\hat{b}_{hq}^{P}$ represent the regulatory abilities of intraspecies TF regulations in host and pathogen GRNs; $-\hat{d}_{jk}^{H}$ and $-\hat{d}_{hk}^{P}$ denote the repression abilities of host miRNA on host gene and pathogen gene, respectively; $\hat{e}_{jn}^{H}$ and $\hat{x}_{j\left(I_j^{''}\left(i^{'}-1\right)+i^{''}\right)}^{H}$ indicate the regulatory abilities of host lncRNA and host complex with regard to host gene in the intraspecies host GRN; $\hat{y}_{ki}^{H}$ and $\hat{z}_{ni}^{H}$ represent the regulatory abilities of host TF on host miRNA and lncRNA in the host intraspecies GRN, respectively. All these weighted connections (links) constitute the combined network matrix *H*. Note that if a connection has been removed by AIC or was not built in candidate GEIN by big data mining, the corresponding location in matrix *H* would be set to zero. We then extracted the core components of GEIN using PNP method, which is a principal network structure projection method based on the principal singular values in the reduction of network dimension by deleting insignificant structures. Therefore, the combined network matrix *H* can be represented by singular value decomposition form as follows,

$$H = U \times D \times V^T \tag{S22}$$

where $U \in \mathbb{R}^{(2I+2Q+K+N)\times\left(I+Q+K+N+I^{'}I^{''}\right)}$; $V \in \mathbb{R}^{\left(I+Q+K+N+I^{'}I^{''}\right)\times\left(I+Q+K+N+I^{'}I^{''}\right)}$; and $D = \mathbf{diag}\left(d_1,\ldots,d_s,\ldots,d_{I+Q+K+N+I^{'}I^{''}}\right)$ is the diagonal matrix of $d_1,\ldots,d_{I+Q+K+N+I^{'}I^{''}}$, which contains $I+Q+K+N+I^{'}I^{''}$ singular values of the combined network matrix *H* with a descending order, that is, $d_1 \geq \ldots \geq d_s \geq \ldots \geq d_{I+Q+K+N+I^{'}I^{''}}$. In addition, we defined the Eigenexpression fraction (E_s) as the following normalization form,

$$E_s = \frac{d_s^2}{\sum_{s=1}^{I+Q+K+N+I'I''} d_s^2} \tag{S23}$$

To guarantee the integrality of the network structure, we select the minimum S such that $\sum_{s=1}^{S} E_S \geq 0.85$, that is, the top S principal components containing 85% network structure of GEIN from the energy perspective. Therefore, the projection of H to the top S singular vectors of U and V are defined, respectively, as follows,

$$\begin{gathered} V_L(w_L,s) = h_{:,w_L}^T \times u_{:,s} \text{ and } V_R(w_R,s) = h_{w_R,:} \times v_{:,s} \\ \text{for } w_L = 1,\ldots,I+Q+K+N+I'I'', \\ w_R = 1,\ldots,2I+2Q+K+N \text{ and } s = 1,\ldots,S \end{gathered} \tag{S24}$$

where $h_{:,w_L}$, $h_{w_R,:}$, $u_{:,s}$ and $v_{:,s}$ represent the w_Lth column of H, the w_Rth row of H, the sth column of U, and the sth row of V, respectively. We further defined the 2-norm projection value of each node (protein/gene/miRNA/lncRNA/complex) in GEIN to the top S left-singular vectors and right-singular vectors as follows,

$$\begin{gathered} D_L(w_L) = \left[\sum_{s=1}^{S}\left[V_L(w_L,s)\right]^2\right]^{1/2} \\ D_R(w_R) = \left[\sum_{s=1}^{S}\left[V_R(w_R,s)\right]^2\right]^{1/2}, \\ \text{for } w_L = 1,\ldots,I+Q+K+N+I'I'' \text{ and } w_R = 1,\ldots,2I+2Q+K+N \end{gathered} \tag{S25}$$

The physical meaning of (S25) is that if the projection value $D_L(w_L)$ is close to zero, the corresponding w_Lth node is almost independent to the core network reconstructed by the top S singular vectors; The larger the projection value, the larger the contribution of the node to the core network. So does the relationship between $D_R(w_R)$ and the w_Rth node. Finally, we can extract the host-pathogen core networks (HPNs) from the GEIN of the early and late stage of CDI, respectively, by assessing the projection value of each node in (S25).

Since the purpose of this study is to identify the cross-talk mechanisms that contribute to the progression of CDI, we targeted the core host/pathogen proteins with top projection values, and their connecting TF/miRNA/lncRNA/complex to form the HPN for further systematic investigation.

Multi-molecule drug design for treating CDI

Currently, the major agents used to treat CDI include metronidazole, vancomycin, and fidaxomicin. In patients with mild symptoms, the guidelines from the Society of Healthcare Epidemiology of America (SHEA) and the Infectious Diseases Society of America (IDSA) recommend treating CDI with metronidazole and vancomycin. However, both antibiotics result in side effects and the recurrence rate still remains unacceptably high (44). A meta-analysis shows that the recurrence rate of CDI is between 13% and 50% of all patients after an initial episode (45). In addition, vancomycin and fidaxomicin are the only two FDA approved drugs used to treat CDI, and the corresponding relapse rates are 24% and 13%, respectively (46). Most recurrent cases are induced by spore-mediated re-infection, suggesting the importance of endospores in CDI.

After predicting these potential multi-molecule drug targets, we then performed drug mining from databases and literature review to design a multi-molecule drug that targets these potential drug targets. Since there is currently no existing drug database for drugs targeting *C. difficile* proteins, we explored studies that predict drugs inhibiting these potential drug targets. One study showed that CD2787 (Cwp84) is a cysteine protease (27), the specific cysteine inhibitor E64 can repress the activity of CD2787 and its degrading-ability toward ECM proteins (27). Another report has suggested that chicken-produced

protein-specific egg yolk antibodies (IgY) can be considered as a potential therapy of CDI (47). These protein-specific antibodies affect *C. difficile* surface proteins including CD2787, CD0239, and CD0237, especially the purified CD0237-specific IgY. Furthermore, REP 3123 dihydrochloride (REP3123) has been reported to inhibit toxin production and spore formation in *C. difficile*, thereby reducing pathogenesis in a hamster model (48). In the case of host cells, we applied the host core network biomarkers of HPNs to the database extracted from the Connectivity Map (CMap) (49). CMap contains the genome-wide expression data of five cell lines (HL60, MCF7, PC3, SKMEL5, and ssMCF7) in response to 1,327 drugs. Since Caco-2 and MCF7 are epithelial cells, we adopted the database of MCF7 cells in CMap for further correlation computation. Here, we aimed to increase the expression of dysfunction proteins (RHOA, CDC42, RAC1, HSP90B1, HSPA5, and HSP90B2P) and repress the expression of inflammation-related proteins (NFKB1, REL and IL-8) after administration of the drugs. The full table of potential drugs and the corresponding correlation values are listed in supplementary S2 Table. Drugs (camptothecin and apigenin) with top correlation values that satisfy all criteria (positive for dysfunction proteins and negative for inflammation-related proteins), were thus selected as potential drugs for treating the cytopathic effects induced by *C. difficile*. In addition to the regulatory ability of the above genes, camptothecin has anticancer, antiviral, and antifungal properties. A recent study also investigated the antibacterial ability of camptothecin, highlighting the potential antibiotic usage of this drug (50). Similarly, as a natural product derived from many plants, apigenin has also been reported as a novel anti-resistance antibiotic (51). The combination of camptothecin and apigenin can enhance the expression of dysfunction proteins, inhibit inflammation-related proteins, and confer antimicrobial

abilities against *C. difficile*.

Finally, we combined these drugs as the potential multi-molecule drug shown in S5 Fig for the predicted multiple drug targets. This multi-molecule drug could induce efficient prevention and elimination of *C. difficile* and remedial effects to restore gene expression homeostasis. The cysteine protease inhibitor E64 and CD0237-specific IgY can inhibit the activities of CD2787 and CD0237, thus interfering with cell adhesion and cell surface protein maturation (27). In addition, according to our results, the inhibition of CD2787 and CD0237 will limit the toxin production and the formation of biofilm, reducing not only the probability of cell adhesion but also the cytotoxicity of *C. difficile*. REP3123 can repress the spore formation and toxin production of *C. difficile*. The repressed toxin production could limit pathogenesis progression and the inhibition of sporulation could prevent spore-mediated re-infection. Furthermore, the combination of human drugs (camptothecin and apigenin) can promote the expression of dysfunctional proteins (RHOA, CDC42, RAC1, HSP90B1, HSPA5, and HSP90B2P) and repress inflammation-related proteins (NFKB1, REL and IL-8) against the severe pathogenic effects induced by *C. difficile*. They could also provide potential antibiotic activity based on recent studies.

S1 Table. List of previously reported essential genes of *C. difficile* in HPNs

	Identified *C. difficile* essential genes of the HPCNs
Early stage	CD2664[3], CD2335[3], CD3550[3], CD0198[3], CD1225[1], CD0130[1], CD0123[3], CD0067[2], CD3540[2], CD1255[2], CD2714[2], CD3256[2], CD1316[2], CD0095[2] and CD2739[2]
Late stage	CD2588[3], CD1816[1], CD0130[1], CD3170[2], CD2744[2], CD2771[2], CD2462[2], CD3304[2], CD2781[2], CD3540[2], CD1145[2], CD2461[2], CD2793[2], CD0059[2], CD1275[2], CD0052[2] and CD1767[2]

[1]Genes represent the *C. difficile* essential genes identified by FBA and SA.
[2]Genes denote the *C. difficile* essential genes identified by TraDIS.
[3]Genes signify the *C. difficile* essential genes identified by FBA, SA and TraDIS.

S2 Table. The full table of potential drugs and the corresponding correlation values.

	carcinine	carbarsone	carisoprodol	dl-alpha tocopherol	apigenin	ginkgolide A	lysergol	piperine	pepstatin	diazoxide	clindamycin	nicergoline
HSPA5	0.06	0.08	0.09	0.10	0.15	0.05	0.04	0.03	0.05	0.06	0.03	0.04
RHOA	0.08	0.09	0.14	0.12	0.19	0.04	0.03	0.06	0.08	0.08	0.05	0.08
CDC42	0.02	0.04	0.06	0.03	0.07	0.05	0.03	0.02	0.05	0.02	0.04	0.04
RAC1	0.15	0.09	0.10	0.10	0.15	0.09	0.03	0.11	0.13	0.06	0.04	0.08
HSP90B1	0.08	0.09	0.13	0.11	0.14	0.10	0.02	0.07	0.10	0.06	0.04	0.10
HSP90B2P	0.02	0.09	0.14	0.16	0.20	0.15	0.07	0.19	0.22	0.08	0.05	0.11
NFKB1	-0.03	-0.05	-0.03	-0.05	-0.04	-0.04	-0.08	-0.04	-0.05	-0.02	-0.04	-0.0
REL	-0.04	-0.04	-0.05	-0.04	-0.04	-0.04	-0.05	-0.04	-0.01	-0.06	-0.05	-0.05
IL-8	-0.02	-0.02	-0.07	-0.06	-0.07	-0.04	-0.06	-0.02	0	-0.09	-0.12	-0.03
	hexamethonium bromide	sulfaguanidine	hydroxyzine	procaine	benzonatate	imipramine	trimetazidine	noscapine	monocrotaline	dacarbazine	cytisine	hydrocotarnine
HSPA5	0.03	0.02	0.11	0.03	0.05	0.09	0	0.12	0.09	0.04	0.07	0.13
RHOA	0.06	0.05	0.10	0.06	0.09	0.07	0.01	0.11	0.09	0.04	0.08	0.12
CDC42	0.01	0.05	0.01	0.04	0.08	0.05	0	0.07	0.05	0	0.05	0.07
RAC1	0.03	0.10	0.08	0.02	0.05	0.03	0.03	0.05	0.06	0.03	0.05	0.07
HSP90B1	0.05	0.02	0.08	0.04	0.09	0.10	0.01	0.11	0.07	0.02	0.06	0.09
HSP90B2P	0.06	0.14	0.08	0.03	0.07	0.04	0.05	0.07	0.06	0.02	0.07	0.07
NFKB1	-0.06	0	0	-0.05	-0.02	-0.04	-0.04	0	-0.03	-0.05	-0.03	-0.01
REL	-0.05	-0.03	0	-0.01	0	-0.03	-0.04	-0.01	-0.04	-0.06	-0.02	-0.04
IL-8	-0.06	-0.01	-0.02	0	-0.02	-0.03	-0.04	-0.08	-0.09	-0.08	-0.03	-0.09

gallamine triethiodide	spironolactone	dihydrostreptomycin	piroxicam	etodolac	cloxacillin	doxylamine	pyrimethamine	oxybuprocaine
0.04	0.03	0.02	0.06	0.02	0.02	0.02	0.03	0.09
0.10	0.08	0.09	0.08	0.07	0.07	0.06	0.04	0.10
0.04	0.06	0.01	0	0	0.04	0.06	0.04	0.05
0.09	0.08	0.10	0.06	0.06	0.07	0.14	0.11	0.15
0.10	0.06	0.07	0.09	0.07	0.09	0.03	0.03	0.11
0.11	0.09	0.12	0.07	0.06	0.10	0.16	0.16	0.15
-0.01	0	-0.01	-0.02	-003	0	-0.01	-0.05	0
-0.05	-0.05	-0.05	-0.07	-0.07	-0.07	0	-0.02	0
-0.05	-0.05	-0.05	-0.11	-0.09	-0.12	0	0	-0.01
acepromazine	ursolic acid	palmatine	harmaline	dydrogesterone	picrotoxinin	salbutamol	camptothecin	flavoxate
0.12	0.06	0.08	0.13	0.08	0.07	0.10	0.13	0.04
0.09	0.07	0.10	0.15	0.05	0.07	0.12	0.26	0.08
0.05	0.05	0.04	0.08	0.06	0.06	0.04	0.09	0.05
0.04	0.03	0.05	0.11	0.08	0.09	0.13	0.18	0.04
0.08	0.04	0.11	0.18	0.07	0.04	0.09	0.21	0.08
0.02	0.03	0.07	0.14	0.12	0.12	0.14	0.23	0.03
0	-0.06	-0.01	-0.01	-0.01	-0.01	-0.01	-0.01	-0.06
-0.04	-0.05	-0.01	-0.01	-0.01	0	-0.01	-0.11	-0.01
-0.06	-0.11	-0.07	-0.04	-0.02	-0.02	-0.02	-0.04	-0.05

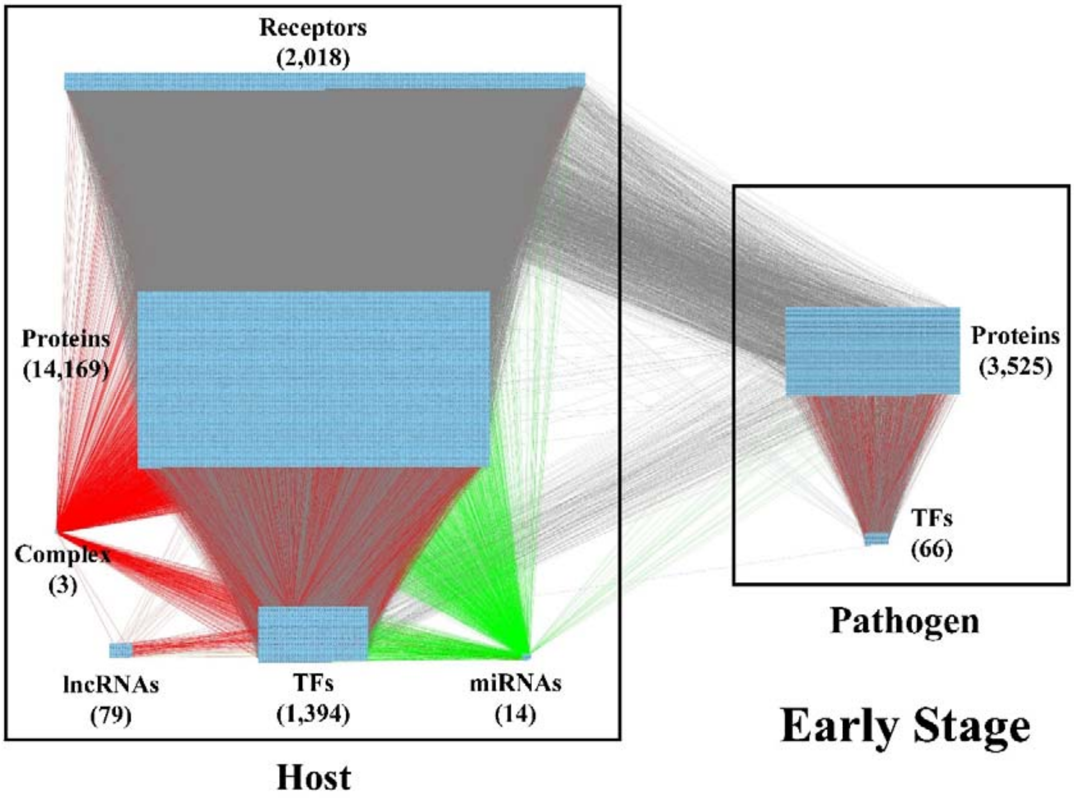

S1 Fig. The real genome-wide GEIN of Caco-2 cells among all three replicates during the early stage of CDI. The real GEINs in Fig 2 of all three replicates during the early-stage of CDI are combined with in this figure. The grey lines represent the protein-protein interaction; the red lines denote the transcriptional regulation; and the green lines signify the miRNA repression.

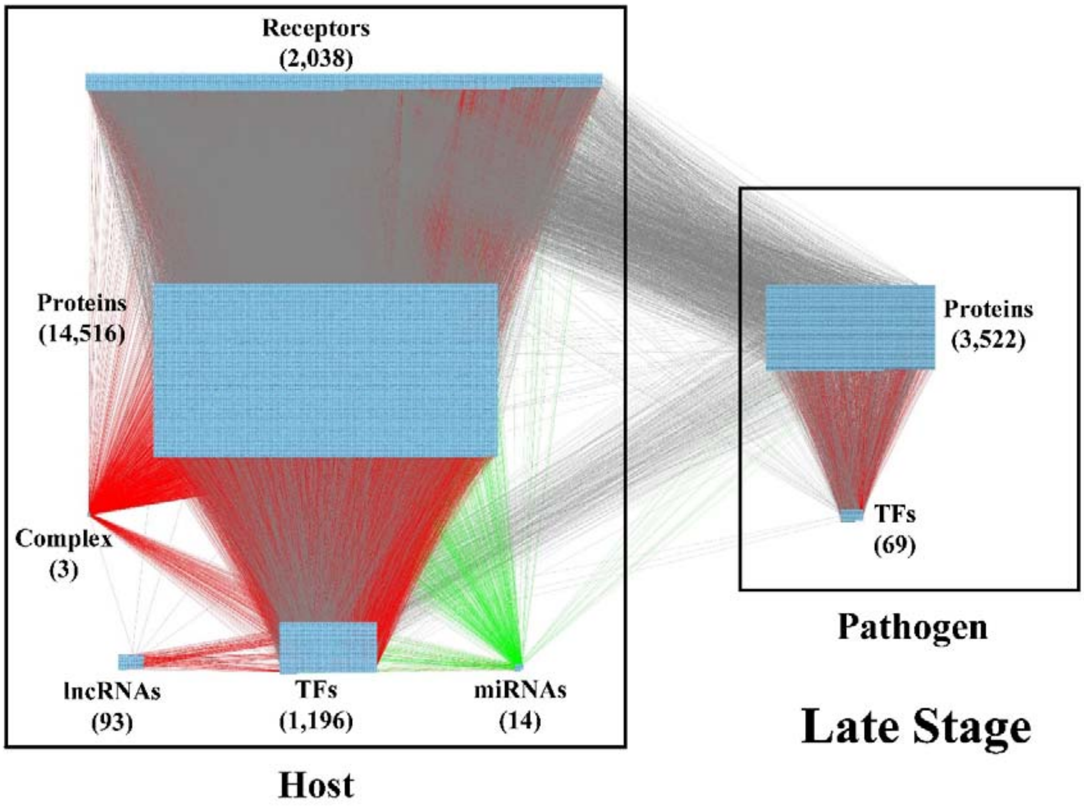

S2 Fig. The real genome-wide GEIN of Caco-2 cells among all three replicates during the late stage of CDI. The real GEINs in Fig 2 of all three replicates during the late-stage of CDI are combined with in this figure. The grey lines represent the protein-protein interaction; the red lines denote the transcriptional regulation; and the green lines signify the miRNA repression.

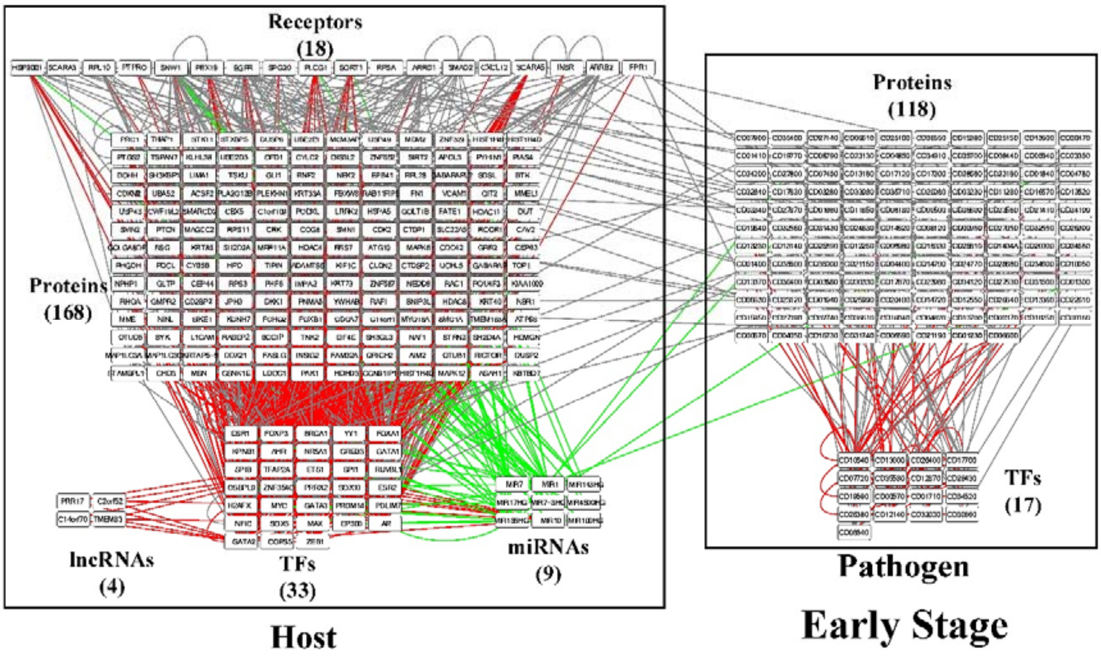

S3 Fig. HPN of Caco-2 cells during the early-stage of CDI. This HPN was extracted from the real GEIN in S1 Fig by the proposed PNP method. The grey lines represent the protein-protein interaction; the red lines denote the transcriptional regulation; and the green lines signify the miRNA repression.

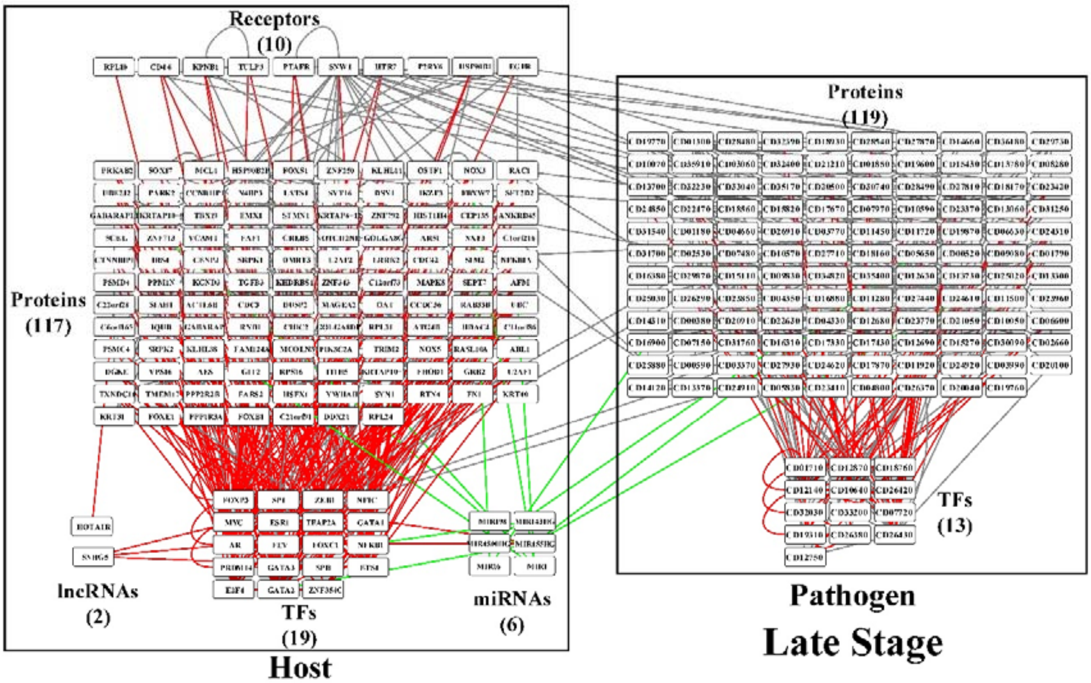

S4 Fig. HPN of Caco-2 cells during the late-stage of CDI. This HPN was extracted from the real GEIN in S2 Fig by the proposed PNP method. The grey lines represent the protein-

protein interaction; the red lines denote the transcriptional regulation; and the green lines signify the miRNA repression.

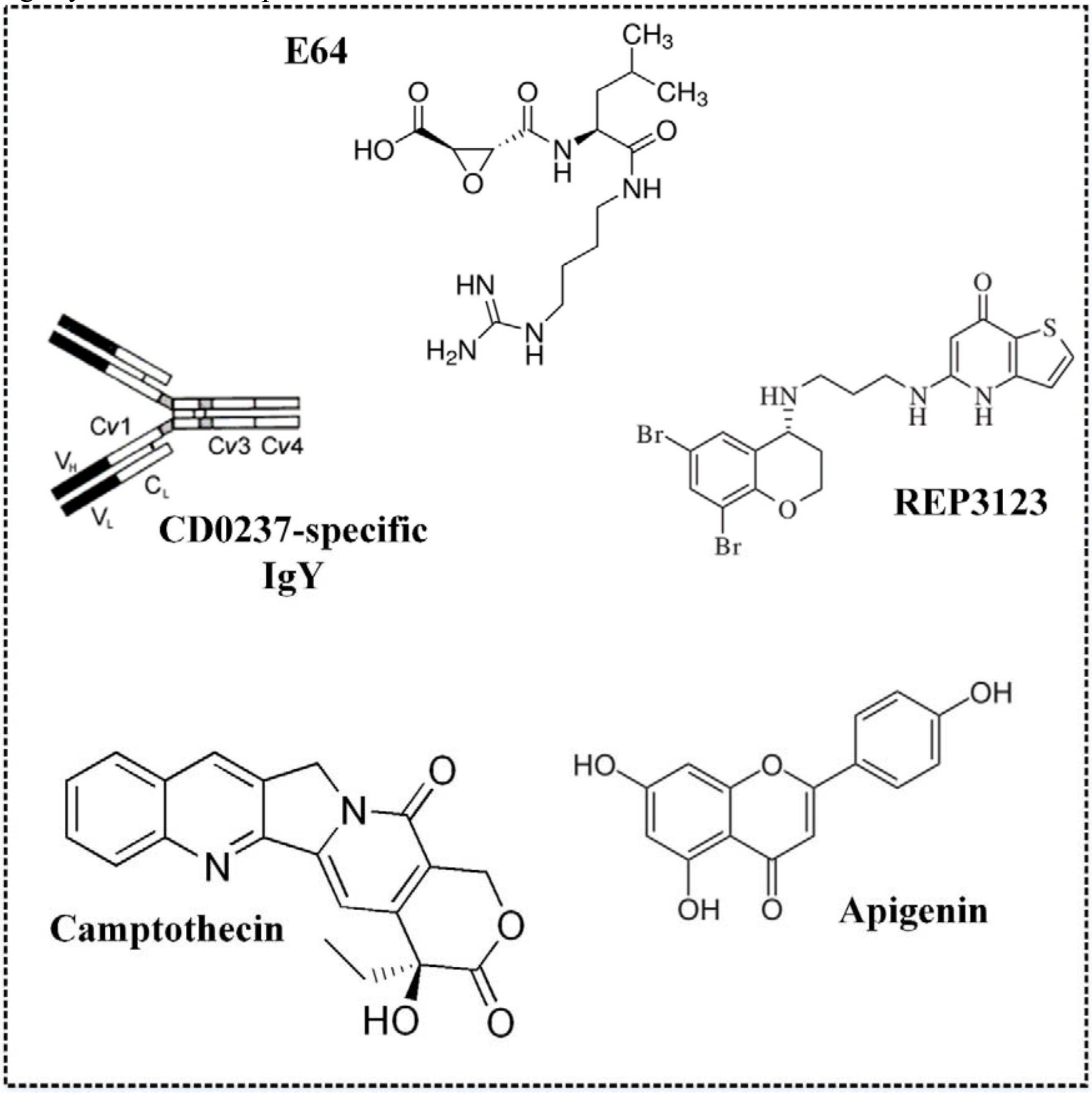

S5 Fig. The potential multi-molecule drug for treatment of *C. difficile*. The E64 inhibitor and the CD0237-specific IgY can inhibit the activities of cell surface proteins CD2787 and CD0237 of *C. difficile*, and REP3123 can repress the toxin production and spore formation of *C. difficile*. The combination of camptothecin and apigenin can thus up-regulate the expression of dysfunction proteins and down-regulate the inflammation- and apoptosis-related proteins.

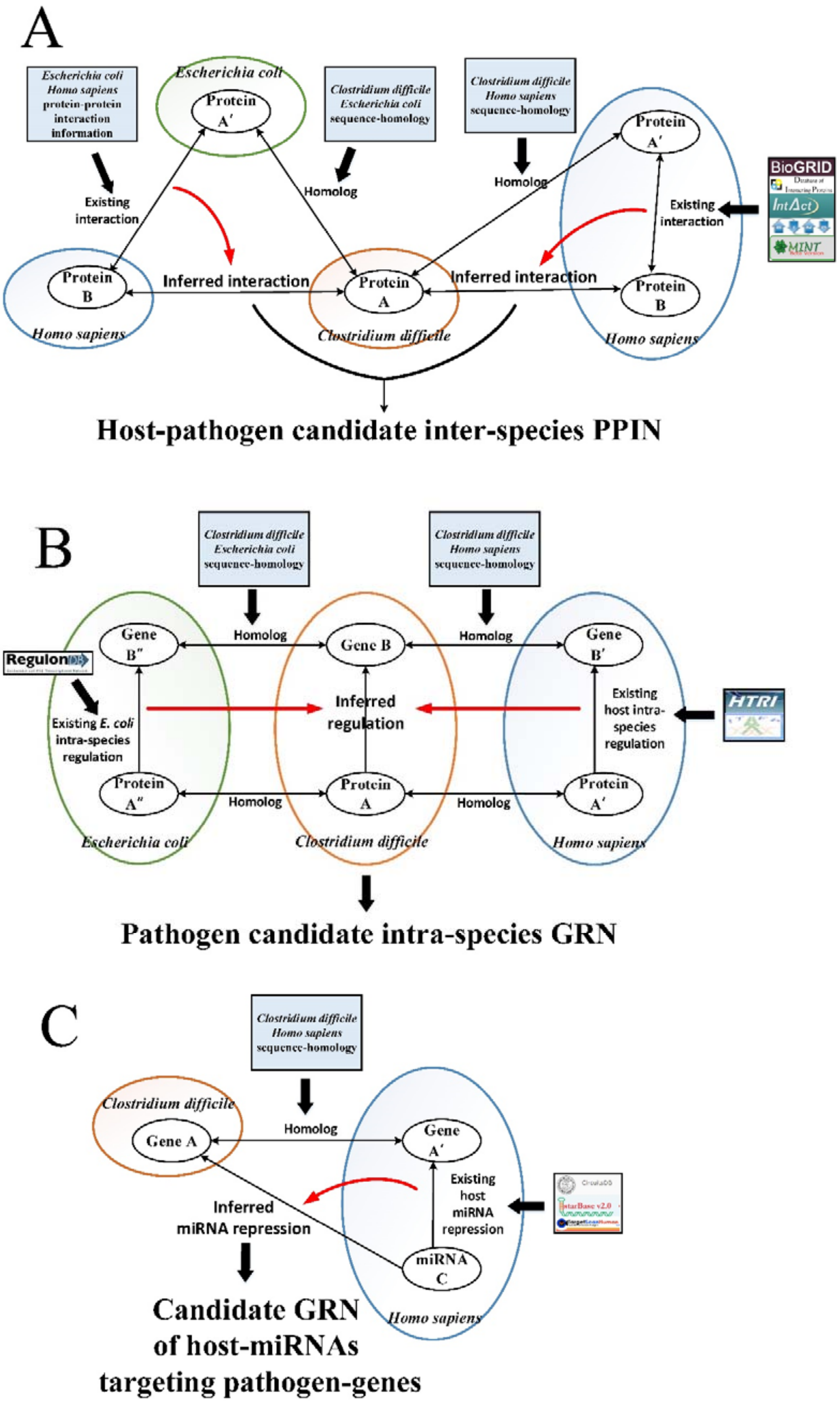
A
Escherichia coli
Homo sapiens
protein-protein
interaction
information
Escherichia coli
Protein
A′
Clostridium difficile
Escherichia coli
sequence-homology
Clostridium difficile
Homo sapiens
sequence-homology
Protein
A′
Existing
interaction
Homolog
Homolog
Existing
interaction
BioGRID
IntAct
MINT
Protein
B
Inferred interaction
Protein
A
Inferred interaction
Protein
B
Homo sapiens
Clostridium difficile
Homo sapiens
Host-pathogen candidate inter-species PPIN
B
Clostridium difficile
Escherichia coli
sequence-homology
Clostridium difficile
Homo sapiens
sequence-homology
Gene
B″
Homolog
Gene B
Homolog
Gene
B′
RegulonDB
Existing E. coli
intra-species
regulation
Inferred
regulation
Existing
host intra-
species
regulation
HTRI
Protein
A″
Homolog
Protein
A
Homolog
Protein
A′
Escherichia coli
Clostridium difficile
Homo sapiens
Pathogen candidate intra-species GRN
C
Clostridium difficile
Homo sapiens
sequence-homology
Clostridium difficile
Gene A
Homolog
Gene
A′
Inferred
miRNA repression
Existing
host
miRNA
repression
CircuitsDB
starBase v2.0
miRNA
C
Homo sapiens
Candidate GRN
of host-miRNAs
targeting pathogen-genes

S6 Fig. The constructing schemes of candidate GEIN. A) Host-pathogen candidate inter-species PPIN; B) pathogen candidate intra-species GRN; and C) host-pathogen interspecies candidate GRN. The sequence-homolog pairs between *C. difficile* and *E. coli*, and *C. difficile* with *homo sapiens*, are satisfied with the standard criteria of sequence-homology (E-value < 10^{-5}, Identity > 30%, Overlap > 80%). With the help of existing host intraspecies PPIN and human-*E. coli* interspecies PPIN, we can infer potential human-*C. difficile* interspecies PPIN as shown in (A); similarly, we can infer potential pathogen intraspecies GRN via sequence-homology, HTRIdb, ITFP and Regulon databases in (B); and in (C), the candidate GRN of host miRNA targeting pathogen gene can be also inferred from TargetScanHuman, starBase v2 and CircuitDB.

Reference

1. X. Wang, Y. Yamamoto, L. Wilson, T. Zhang, B. Howitt, G. Ning, Y. Hong, C. C. Khor, B. Chevalier, F. A. Sylvester, M. Farrow, B. Lacy, K. Y. Ho, C. Crum, F. McKeon and W. Xian: Cloning and Variation of Ground State Intestinal Stem Cells. *Gastroenterology*, 148(4), S729-S729 (2015)
2. T. Janvilisri, J. Scaria and Y. F. Chang: Transcriptional Profiling of Clostridium difficile and Caco-2 Cells during Infection. *Journal of Infectious Diseases*, 202(2), 282-290 (2010) doi:10.1086/653484
3. L. Salwinski, C. S. Miller, A. J. Smith, F. K. Pettit, J. U. Bowie and D. Eisenberg: The Database of Interacting Proteins: 2004 update. *Nucleic Acids Research*, 32, D449-D451 (2004) doi:10.1093/nar/gkh086
4. G. D. Bader, D. Betel and C. W. V. Hogue: BIND: the Biomolecular Interaction Network Database. *Nucleic Acids Research*, 31(1), 248-250 (2003) doi:10.1093/nar/gkg056
5. S. Orchard, M. Ammari, B. Aranda, L. Breuza, L. Briganti, F. Broackes-Carter, N. H. Campbell, G. Chavali, C. Chen, N. del-Toro, M. Duesbury, M. Dumousseau, E. Galeota, U. Hinz, M. Iannuccelli, S. Jagannathan, R. Jimenez, J. Khadake, A. Lagreid, L. Licata, R. C. Lovering, B. Meldal, A. N. Melidoni, M. Milagros, D. Peluso, L. Perfetto, P. Porras, A. Raghunath, S. Ricard-Blum, B. Roechert, A. Stutz, M. Tognolli, K. van Roey, G. Cesareni and H. Hermjakob: The MIntAct project-IntAct as a common curation platform for 11 molecular interaction databases. *Nucleic Acids Research*, 42(D1), D358-D363 (2014) doi:10.1093/nar/gkt1115
6. L. Licata, L. Briganti, D. Peluso, L. Perfetto, M. Iannuccelli, E. Galeota, F. Sacco, A. Palma, A. P. Nardozza, E. Santonico, L. Castagnoli and G. Cesareni: MINT, the molecular interaction database: 2012 update. *Nucleic Acids Research*, 40(D1), D857-D861 (2012) doi:10.1093/nar/gkr930
7. A. Chatr-aryamontri, B. J. Breitkreutz, R. Oughtred, L. Boucher, S. Heinicke, D. C. Chen, C. Stark, A. Breitkreutz, N. Kolas, L. O'Donnell, T. Reguly, J. Nixon, L. Ramage, A. Winter, A. Sellam, C. Chang, J. Hirschman, C. Theesfeld, J. Rust, M. S. Livstone, K. Dolinski and M. Tyers: The BioGRID interaction database: 2015 update. *Nucleic Acids Research*, 43(D1), D470-D478 (2015) doi:10.1093/nar/gku1204
8. L. A. Bovolenta, M. L. Acencio and N. Lemke: HTRIdb: an open-access database for experimentally verified human transcriptional regulation interactions. *Bmc*

Genomics, 13 (2012) doi:Artn 405
10.1186/1471-2164-13-405
9. G. Y. Zheng, K. Tu, Q. Yang, Y. Xiong, C. C. Wei, L. Xie, Y. Y. Zhu and Y. X. Li: ITFP: an integrated platform of mammalian transcription factors. *Bioinformatics*, 24(20), 2416-2417 (2008) doi:10.1093/bioinformatics/btn439
10. V. Agarwal, G. W. Bell, J. W. Nam and D. P. Bartel: Predicting effective microRNA target sites in mammalian mRNAs. *Elife*, 4 (2015) doi:ARTN e05005
10.7554/eLife.05005
11. J. H. Li, S. Liu, H. Zhou, L. H. Qu and J. H. Yang: starBase v2.0: decoding miRNA-ceRNA, miRNA-ncRNA and protein-RNA interaction networks from large-scale CLIP-Seq data. *Nucleic Acids Research*, 42(D1), D92-D97 (2014) doi:10.1093/nar/gkt1248
12. O. Friard, A. Re, D. Taverna, M. De Bortoli and D. Cora: CircuitsDB: a database of mixed microRNA/transcription factor feed-forward regulatory circuits in human and mouse. *Bmc Bioinformatics*, 11 (2010) doi:Artn 435
10.1186/1471-2105-11-435
13. D. Szklarczyk, A. Franceschini, S. Wyder, K. Forslund, D. Heller, J. Huerta-Cepas, M. Simonovic, A. Roth, A. Santos, K. P. Tsafou, M. Kuhn, P. Bork, L. J. Jensen and C. von Mering: STRING v10: protein-protein interaction networks, integrated over the tree of life. *Nucleic Acids Research*, 43(D1), D447-D452 (2015) doi:10.1093/nar/gku1003
14. D. E. Voth and J. D. Ballard: Clostridium difficile toxins: Mechanism of action and role in disease. *Clinical Microbiology Reviews*, 18(2), 247-+ (2005) doi:10.1128/Cmr.18.2.247-263.2005
15. M. E. LaFrance, M. A. Farrow, R. Chandrasekaran, J. S. Sheng, D. H. Rubin and D. B. Lacy: Identification of an epithelial cell receptor responsible for Clostridium difficile TcdB-induced cytotoxicity. *Proceedings of the National Academy of Sciences of the United States of America*, 112(22), 7073-7078 (2015) doi:10.1073/pnas.1500791112
16. P. F. Yuan, H. M. Zhang, C. Z. Cai, S. Y. Zhu, Y. X. Zhou, X. Z. Yang, R. N. He, C. Li, S. J. Guo, S. Li, T. X. Huang, G. Perez-Cordon, H. P. Feng and W. S. Wei: Chondroitin sulfate proteoglycan 4 functions as the cellular receptor for Clostridium difficile toxin B. *Cell Research*, 25(2), 157-168 (2015) doi:10.1038/cr.2014.169
17. P. Papatheodorou, C. Zamboglou, S. Genisyuerek, G. Guttenberg and K. Aktories: Clostridial Glucosylating Toxins Enter Cells via Clathrin-Mediated Endocytosis. *Plos One*, 5(5) (2010) doi:ARTN e10673
10.1371/journal.pone.0010673
18. X. Na, H. Kim, M. P. Moyer, C. Pothoulakis and J. T. LaMont: gp96 is a human colonocyte plasma membrane binding protein for Clostridium difficile toxin A. *Infection and Immunity*, 76(7), 2862-2871 (2008) doi:10.1128/Iai.00326-08
19. G. Haug, K. Aktories and H. Barth: The host cell chaperone Hsp90 is necessary for cytotoxic action of the binary iota-like toxins. *Infection and Immunity*, 72(5), 3066-3068 (2004) doi:10.1128/Iai.72.5.3066-3068.2004
20. S. D. Goy, A. Olling, D. Neumann, A. Pich and R. Gerhard: Human neutrophils are activated by a peptide fragment of Clostridium difficile toxin B presumably via formyl peptide receptor. *Cellular Microbiology*, 17(6), 893-909 (2015) doi:10.1111/cmi.12410
21. E. ChavesOlarte, M. Weidmann, C. vonEichelStreiber and M. Thelestam: Toxins A

and B from Clostridium difficile differ with respect to enzymatic potencies, cellular substrate specificities, and surface binding to cultured cells. *Journal of Clinical Investigation*, 100(7), 1734-1741 (1997) doi:Doi 10.1172/Jci119698
22. B. Berwin, J. P. Hart, S. Rice, C. Gass, S. V. Pizzo, S. R. Post and C. V. Nicchitta: Scavenger receptor-A mediates gp96/GRP94 and calreticulin internalization by antigen-presenting cells. *Embo Journal*, 22(22), 6127-6136 (2003) doi:Doi 10.1093/Emboj/Cdg572
23. T. S. Liu, M. W. Musch, K. Sugi, M. M. Walsh-Reitz, M. J. Ropeleski, B. A. Hendrickson, C. Pothoulakis, J. T. Lamont and E. B. Chang: Protective role of HSP72 against Clostridium difficile toxin A-induced intestinal epithelial cell dysfunction. *American Journal of Physiology-Cell Physiology*, 284(4), C1073-C1082 (2003) doi:10.1152/ajpcell.00134.2002
24. K. D. Tucker and T. D. Wilkins: Toxin-a of Clostridium-Difficile Binds to the Human Carbohydrate Antigens-I, Antigens-X, and Antigens-Y. *Infection and Immunity*, 59(1), 73-78 (1991)
25. H. Kim, S. H. Rhee, C. Pothoulakis and J. T. LaMont: Clostridium difficile toxin A binds colonocyte Src causing dephosphorylation of focal adhesion kinase and paxillin. *Experimental Cell Research*, 315(19), 3336-3344 (2009) doi:10.1016/j.yexcr.2009.05.020
26. E. Calabi, F. Calabi, A. D. Phillips and N. F. Fairweather: Binding of Clostridium difficile surface layer proteins to gastrointestinal tissues. *Infection and Immunity*, 70(10), 5770-5778 (2002) doi:10.1128/Iai.70.10.5770-5778.2002
27. C. Janoir, S. Pechine, C. Grosdidier and A. Collignon: Cwp84, a surface-associated protein of Clostridium difficile, is a cysteine protease with degrading activity on extracellular matrix proteins. *Journal of Bacteriology*, 189(20), 7174-7180 (2007) doi:10.1128/Jb.00578-07
28. M. Larocque, T. Chenard and R. Najmanovich: A curated C-difficile strain 630 metabolic network: prediction of essential targets and inhibitors. *Bmc Systems Biology*, 8 (2014) doi:Artn 117
10.1186/S12918-014-0117-Z
29. O. Krishnadev and N. Srinivasan: Prediction of protein-protein interactions between human host and a pathogen and its application to three pathogenic bacteria. *International Journal of Biological Macromolecules*, 48(4), 613-619 (2011) doi:10.1016/j.ijbiomac.2011.01.030
30. A. Antunes, I. Martin-Verstraete and B. Dupuy: CcpA-mediated repression of Clostridium difficile toxin gene expression. *Molecular Microbiology*, 79(4), 882-899 (2011) doi:10.1111/j.1365-2958.2010.07495.x
31. I. El Meouche, J. Peltier, M. Monot, O. Soutourina, M. Pestel-Caron, B. Dupuy and J. L. Pons: Characterization of the SigD Regulon of C. difficile and Its Positive Control of Toxin Production through the Regulation of tcdR. *Plos One*, 8(12) (2013) doi:UNSP e83748
10.1371/journal.pone.0083748
32. S. Matamouros, P. England and B. Dupuy: Clostridium difficile toxin expression is inhibited by the novel regulator TcdC. *Molecular Microbiology*, 64(5), 1274-1288 (2007) doi:10.1111/j.1365-2958.2007.05739.x

33. K. A. Fimlaid, J. P. Bond, K. C. Schutz, E. E. Putnam, J. M. Leung, T. D. Lawley and A. Shen: Global Analysis of the Sporulation Pathway of Clostridium difficile. *Plos Genetics*, 9(8) (2013) doi:ARTN e1003660
10.1371/journal.pgen.1003660
34. A. Antunes, E. Camiade, M. Monot, E. Courtois, F. Barbut, N. V. Sernova, D. A. Rodionov, I. Martin-Verstraete and B. Dupuy: Global transcriptional control by glucose and carbon regulator CcpA in Clostridium difficile. *Nucleic Acids Research*, 40(21), 10701-10718 (2012) doi:10.1093/nar/gks864
35. P. S. Novichkov, A. E. Kazakov, D. A. Ravcheev, S. A. Leyn, G. Y. Kovaleva, R. A. Sutormin, M. D. Kazanov, W. Riehl, A. P. Arkin, I. Dubchak and D. A. Rodionov: RegPrecise 3.0-A resource for genome-scale exploration of transcriptional regulation in bacteria. *Bmc Genomics*, 14 (2013) doi:Artn 745
10.1186/1471-2164-14-745
36. N. Mani and B. Dupuy: Regulation of toxin synthesis in Clostridium difficile by an alternative RNA polymerase sigma factor. *Proceedings of the National Academy of Sciences of the United States of America*, 98(10), 5844-5849 (2001) doi:DOI 10.1073/pnas.101126598
37. S. S. Dineen, A. C. Villapakkam, J. T. Nordman and A. L. Sonenshein: Repression of Clostridium difficile toxin gene expression by CodY. *Molecular Microbiology*, 66(1), 206-219 (2007) doi:10.1111/j.1365-2958.2007.05906.x
38. L. Saujet, M. Monot, B. Dupuy, O. Soutourina and I. Martin-Verstraete: The Key Sigma Factor of Transition Phase, SigH, Controls Sporulation, Metabolism, and Virulence Factor Expression in Clostridium difficile. *Journal of Bacteriology*, 193(13), 3186-3196 (2011) doi:10.1128/Jb.00272-11
39. B. M. Walter, M. Rupnik, V. Hodnik, G. Anderluh, B. Dupuy, N. Paulic, D. Zgur-Bertok and M. Butala: The LexA regulated genes of the Clostridium difficile. *Bmc Microbiology*, 14 (2014) doi:Artn 88
10.1186/1471-2180-14-88
40. H. Salgado, M. Peralta-Gil, S. Gama-Castro, A. Santos-Zavaleta, L. Muniz-Rascado, J. S. Garcia-Sotelo, V. Weiss, H. Solano-Lira, I. Martinez-Flores, A. Medina-Rivera, G. Salgado-Osorio, S. Alquicira-Hernandez, K. Alquicira-Hernandez, A. Lopez-Fuentes, L. Porron-Sotelo, A. M. Huerta, C. Bonavides-Martinez, Y. I. Balderas-Martinez, L. Pannier, M. Olvera, A. Labastida, V. Jimenez-Jacinto, L. Vega-Alvarado, V. del Moral-Chavez, A. Hernandez-Alvarez, E. Morett and J. Collado-Vides: RegulonDB v8.0: omics data sets, evolutionary conservation, regulatory phrases, cross-validated gold standards and more. *Nucleic Acids Research*, 41(D1), D203-D213 (2013) doi:10.1093/nar/gks1201
41. C. W. Li, Y. L. Lee and B. S. Chen: Genetic-and-Epigenetic Interspecies Networks for Cross-Talk Mechanisms in Human Macrophages and Dendritic Cells during MTB Infection. *Frontiers in Cellular and Infection Microbiology*, 6 (2016) doi:Artn 124
10.3389/Fcimb.2016.00124
42. R. Johansson: System modeling and identification. Prentice-hall, (1993)
43. C. W. Li and B. S. Chen: Network Biomarkers of Bladder Cancer Based on a Genome-Wide Genetic and Epigenetic Network Derived from Next-Generation Sequencing Data. *Disease Markers* (2016) doi:Artn 4149608

10.1155/2016/4149608
44. D. Shah, M. D. Dang, R. Hasbun, H. L. Koo, Z. D. Jiang, H. L. DuPont and K. W. Garey: Clostridium difficile infection: update on emerging antibiotic treatment options and antibiotic resistance. *Expert Review of Anti-Infective Therapy*, 8(5), 555-564 (2010) doi:10.1586/Eri.10.28
45. K. W. Garey, S. Sethi, Y. Yadav and H. L. DuPont: Meta-analysis to assess risk factors for recurrent Clostridium difficile infection. *Journal of Hospital Infection*, 70(4), 298-304 (2008) doi:10.1016/j.jhin.2008.08.012
46. T. J. Louie, M. A. Miller, K. M. Mullane, K. Weiss, A. Lentnek, Y. Golan, S. Gorbach, P. Sears, Y. K. Shue and O.-.-C. S. Grp: Fidaxomicin versus Vancomycin for Clostridium difficile Infection. *New England Journal of Medicine*, 364(5), 422-431 (2011) doi:Doi 10.1056/Nejmoa0910812
47. G. L. Mulvey, T. C. Dingle, L. Fang, J. Strecker and G. D. Armstrong: Therapeutic potential of egg yolk antibodies for treating Clostridium difficile infection. *Journal of Medical Microbiology*, 60(8), 1181-1187 (2011) doi:10.1099/jmm.0.029835-0
48. U. A. Ochsner, S. J. Bell, A. L. O'Leary, T. Hoang, K. C. Stone, C. L. Young, I. A. Critchley and N. Janjic: Inhibitory effect of REP3123 on toxin and spore formation in Clostridium difficile, and in vivo efficacy in a hamster gastrointestinal infection model. *Journal of Antimicrobial Chemotherapy*, 63(5), 964-971 (2009) doi:10.1093/jac/dkp042
49. J. Lamb, E. D. Crawford, D. Peck, J. W. Modell, I. C. Blat, M. J. Wrobel, J. Lerner, J. P. Brunet, A. Subramanian, K. N. Ross, M. Reich, H. Hieronymus, G. Wei, S. A. Armstrong, S. J. Haggarty, P. A. Clemons, R. Wei, S. A. Carr, E. S. Lander and T. R. Golub: The connectivity map: Using gene-expression signatures to connect small molecules, genes, and disease. *Science*, 313(5795), 1929-1935 (2006) doi:10.1126/science.1132939
50. Q. L. Dong, J. Luo, W. Qiu, L. Cai, S. I. Anjum, B. Li, M. S. Hou, G. L. Xie and G. C. Sun: Inhibitory Effect of Camptothecin against Rice Bacterial Brown Stripe Pathogen Acidovorax avenae subsp avenae RS-2. *Molecules*, 21(8) (2016) doi:Artn 978 10.3390/Molecules21080978
51. Y. Morimoto, T. Baba, T. Sasaki and K. Hiramatsu: Apigenin as an anti-quinolone-resistance antibiotic. *International Journal of Antimicrobial Agents*, 46(6), 666-673 (2015) doi:10.1016/j.ijantimicag.2015.09.006
